# Effect of p53 activation on experimental right ventricular hypertrophy

**DOI:** 10.1371/journal.pone.0234872

**Published:** 2020-06-19

**Authors:** Swathi Veeroju, Argen Mamazhakypov, Nabham Rai, Baktybek Kojonazarov, Valerie Nadeau, Sandra Breuils-Bonnet, Ling Li, Norbert Weissmann, Susanne Rohrbach, Steve Provencher, Sébastien Bonnet, Werner Seeger, Ralph Schermuly, Tatyana Novoyatleva

**Affiliations:** 1 Universities of Giessen and Marburg Lung Center (UGMLC), Excellence Cluster Cardio Pulmonary Institute (CPI), Member of the German Center for Lung Research (DZL), Justus-Liebig University Giessen, Giessen, Germany; 2 Institute for Lung Health, Giessen, Germany; 3 Pulmonary Hypertension and Vascular Biology Research Group, Institut Universitaire de Cardiologie et de Pneumologie de Québec, Université Laval, Department of Medicine, Québec, Canada; 4 Institute of Physiology, Justus-Liebig University Giessen, Giessen, Germany; 5 Max Planck Institute for Heart and Lung Research, Bad Nauheim, Germany; Vanderbilt University Medical Center, UNITED STATES

## Abstract

The leading cause of death in Pulmonary Arterial Hypertension (PAH) is right ventricular (RV) failure. The tumor suppressor p53 has been associated with left ventricular hypertrophy (LVH) and remodeling but its role in RV hypertrophy (RVH) is unclear. The purpose of this study was to determine whether pharmacological activation of p53 by Quinacrine affects RV remodeling and function in the pulmonary artery banding (PAB) model of compensated RVH in mice. The effects of p53 activation on cellular functions were studied in isolated cardiomyocytes, cardiac fibroblasts and endothelial cells (ECs). The expression of p53 was examined both on human RV tissues from patients with compensated and decompensated RVH and in mouse RV tissues early and late after the PAB. As compared to control human RVs, there was no change in p53 expression in compensated RVH, while a marked upregulation was found in decompensated RVH. Similarly, in comparison to SHAM-operated mice, unaltered RV p53 expression 7 days after PAB, was markedly induced 21 days after the PAB. Quinacrine induced p53 accumulation did not further deteriorate RV function at day 7 after PAB. Quinacrine administration did not increase EC death, neither diminished EC number and capillary density in RV tissues. No major impact on the expression of markers of sarcomere organization, fatty acid and mitochondrial metabolism and respiration was noted in Quinacrine-treated PAB mice. p53 accumulation modulated the expression of Heme Oxygenase 1 (HO-1) and Glucose Transporter (Glut1) in mouse RVs and in adult cardiomyocytes. We conclude that early p53 activation in PAB-induced RVH does not cause substantial detrimental effects on right ventricular remodeling and function.

## Introduction

Pulmonary arterial hypertension (PAH) is a disease characterized by pulmonary vascular remodeling and increased pulmonary vascular resistance, finally culminating in right ventricular (RV) hypertrophy and failure [1]. RV dysfunction and failure represents a major clinical problem with no existing specific therapies. Clinically, RV failure follows a period of compensation, so called adaptive hypertrophy, which is characterized by maintained myocardial vascularization. In contrast, the transition to maladaptive RV hypertrophy is associated with a decrease in vascularization [2]. In various experimental models of PH associated with RV failure, reduced myocardial vascular density has been proposed to be a critical contributor to the transition from adaptive to maladaptive RV hypertrophy [3–7]. In left ventricular hypertrophy (LVH) model, induced by transaortic constriction (TAC), proper cardiac angiogenesis is controlled by the hypoxia-inducible factor-1 (HIF-1α) thus contributing to adaptive LVH. Importantly, the transition from LVH to left heart failure is characterized by accumulation of the tumor suppressor p53 [8]. The p53 binds to HIF-1a, causing its degradation and constraining its transactivation potency [9, 10]. In the pressure-overload induced LVH, p53 has been demonstrated to contribute to maladaptive myocardial remodeling via inhibition of HIF-1α activity and impairment of cardiac angiogenesis [8]. p53 accumulation is associated with myocardial apoptosis at the end-stage of human heart failure [11]. In a rat model of RV hypertrophy, p53 is induced early after the surgery and gradually decreased in RV myocardium at the later time points [12]. Interestingly, although RVs from maladaptive RV hypertrophy of Monocrotaline (MCT)-induced PAH exhibit elevations in p53 levels, no changes on RV CM apoptosis have been noted [13].

Accumulating evidence suggests that p53 plays a critical role in experimental pulmonary hypertension. Inactivation of p53 by pifithrin-α (an inhibitor of p53 activity) was sufficient to induce PH in rats, associated with anti-apoptotic and pro-proliferative responses in pulmonary vessels [14]. Furthermore, activation of p53 by a cis-imidazoline analog, Nutlin-3a, a stabilizer of p53 and an inhibitor of the mouse double minute 2 homolog (Mdm2)–p53 interaction, reversed experimental PH by a reduction of proliferation of pulmonary artery smooth muscle cells (PASMCs) [15]. The p53 knockout rats exhibit a decrease in PAH development in the SU5416 (vascular endothelial growth factor inhibitor)/hypoxia (Hx) rat model (SuHx) [16]. Recently, p53 has been demonstrated to control global cardiac transcriptomic changes that coordinate the cardiac architecture and contractility, energy metabolism, and oxidative stress response. [17]. In the present study, we tested the effects of p53 activation by the acridine derivative Quinacrine (stabilizes p53 in a Mdm2-independent manner) [18, 19] on RV function and structure in the mouse PAB model of compensated RV hypertrophy.

## Materials and methods

### Human tissue samples

All studies involving human participants were approved by the Laval University and Institute Universitaire de Cardiologie et de Pneumologie de Québec (IUCPQ) biosafety and human ethics committee (CER-20773). All patients or their legal representatives (in case of autopsy, n = 19) gave informed consent before the beginning of the study. Human RV tissues were obtained in collaboration with the Institut Universitaire de Cardiologie et de Pneumologie de Québec site of the Respiratory Health Network tissue bank of the FRQS. Tissue samples were categorized as control RVs (n = 20), compensated RVH (n = 11), or decompensated RVs (n = 11) on the basis of clinical history and the RV functional parameters (Tables 1 and S1).

**Table 1:** Clinical characteristics of the patients.

	Control (n = 20)	CRVH (n = 11)	DRV PAH (n = 11 all PAH)
**Female sex, %**	30	27	82
**Age, y**	47±12	33±13	62±12
**Functional Class, %**			
**I**	50 (n = 1)	…	…
**II**	50 (n = 1)	50 (n = 2)	…
**III**	…	50 (n = 2)	45 (n = 5)
**IV**	…	…	55 (n = 6)
**RVSP, mmHg**	23±6	50±24	84±25
**TAPSE, mmHg**	25±2**	17±3*	14±3
**PAH medication, %**			
**ERA**	…	…	64
**PDE5**	…	…	55
**Epoprostenol**	…	…	9

CRVH designates compensated right ventricular hypertrophy; DRV, decompensated right ventricle; TAPSE, tricuspid annular plane systolic excursion; RVSP, right ventricular systolic pressure; ERA, endothelin receptor antagonist; PAH, pulmonary arterial hypertension; PDE5, phosphodiesterase-5.

**P<0.01 Control vs DRV;

*P<0.05 CRVH vs DRV. Data were analyzed by nonparametric Mann-Whitney test. Values are expressed as mean ±SD.

Human patients with decompensated RVs were distinguished from the patients with compensated RVH and individuals with control RVs based on the tricuspid annular plane systolic excursion (TAPSE). Amongst nineteen autopsy samples obtained for the study, seven belong to the group of control RVs. One and eleven RV autopsies were from groups of compensated and decompensated RVH, respectively. Specific details of the patient characteristics given in the supplementary information (S1 Table).

### Animal model

All *in vivo* procedures were approved by the local animal ethics committee authorities (Regierungspraesidium Giessen). Adult male C57Bl/6J mice (21–24 g body weight) were purchased from Charles River Laboratories (Sulzbach, Germany). Main pulmonary artery (pulmonary truck) banding (PAB) was performed as previously described [20, 21]. Briefly, mice were injected with buprenorphine hydrochloride 0.05 mg/kg body weight (WB) subcutaneously (s.c.) as an analgesic therapy and anesthetized in anesthetic chamber supplied with continuous flow of isoflurane (2.0–3.0% mixed in 100% oxygen). Following anesthesia induction, orotracheal intubation was performed and mice were placed on a pad and mechanically ventilated using a Minivent (Hugo Sachs, Germany). Chest was opened in the second left intercostal space, the pericardium was removed, pulmonary truck was dissected from surrounding tissues and a partially occlusive titanium clip was placed around the pulmonary trunk (Hemoclip; Edward Weck, Research Triangle Park, NC, USA) to a width of 0.3 mm in diameter, which corresponds to approximately 75% occlusion of the luminal diameter, after which the chest was closed and mice were allowed to recover from anesthesia. SHAM control mice were subjected to the same surgery except for application of the titanium clip to the pulmonary truck. Postoperative analgesia was maintained by administration of buprenorphine hydrochloride 0.05 mg/kg s.c. every 24 hours for 3 to 5 days. To exclude potential variabilities, which may arise due to fluctuating hormone levels, only the male mice have been used for the analyses. The echocardiography and the hemodynamics measurements were performed and analyzed in a blinded manner [22].

### Drugs and antibodies

Quinacrine dihydrochloride (Q3251) and Carboxymethylcellulose Sodium (C9481) were purchased from Sigma Aldrich (Missouri, USA). For isolation of the total proteins from RV tissues, cell lysis buffer (Cell Signaling Technology, Massachusetts, USA) was supplied with Halt Protease and Phosphatase Inhibitor Cocktail (78446, Thermo Fisher Scientific, Massachusetts, USA). For subsequent measurement of the protein concentrations, the Bio-Rad DC Protein Assay was applied (Bio-Rad Laboratories, Inc). For western blot analyses, the NuPAGE LDS Sample Buffer (4X) and NuPAGE Sample Reducing Agent (10X) (Novex™ NuPAGE™, Thermo Fisher Scientific, Massachusetts, USA) were utilized. The list of all primary antibodies utilized for provided as a supplementary information.

### Drug treatment

SHAM or PAB surgery was performed on day 0 followed by the treatment with either 1% Carboxymethylcellulose (Placebo) or Quinacrine (10mg/kg body weight) administered every day by oral gavage from day 1 till day 7. The dose of Quinacrine was chosen based on publications that demonstrated activity on p53 expression and function in mouse models of heart hypertrophy [8]. Echocardiographic measurements were performed on day 6. Hemodynamic measurements followed by organ harvesting were performed on day 7.

### Statistical analysis

The data are expressed as means ± SEM. The exact group size (n) for each experimental group/condition is provided and ‘n’ refers to independent values. Statistical analysis was performed with GraphPad Prism 6.0 software (San Diego, CA, USA; RRID: SCR_002798). Represented data from the Fig 6 for collagen expression at mRNA level (*col1a1* and *col3a*) and collagen area (%) were transformed to logit. To determine statistical significance, one-way analysis of variance (ANOVA) followed by Bonferroni post-hoc test for multiple comparisons and unpaired t-test for two group comparisons were used. P values ˂ 0.05 were considered to be statistically significant.

The specific details of the primary cell culture, real-time PCR, western blot analysis, immunofluorescence/immunohistochemistry and collagen staining are summarized in the supplementary information.

## Results

### p53 activation in human decompensated stage hypertrophy

**Fig 1:**
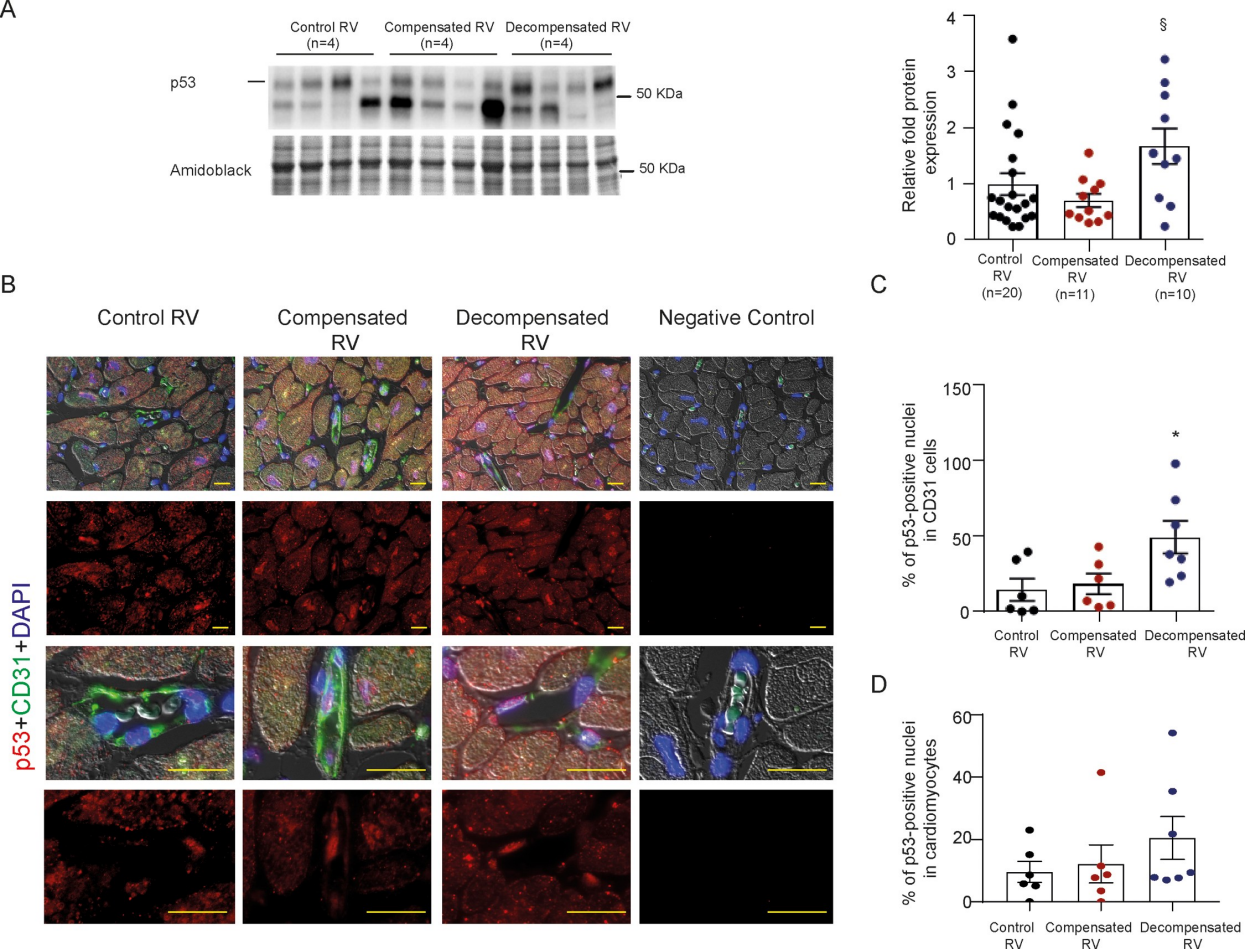
Expression of p53 in compensated and decompensated human right ventricular tissue. (A) Immunoblot analysis of p53 expression and its subsequent densitometric quantification of control (n = 20), compensated (n = 11) and decompensated (n = 10) human right ventricular (RV) tissues. ^§^P < 0.05 decompensated versus compensated. (B) Immunofluorescence images representing p53 expression in CD31-positive cells. Smaller and bigger scale bars indicate 10 and 20μM, respectively. (C) Representative graph demonstrating an increase of p53 expression in CD31-positive cells (in %) in control (n = 6), compensated (n = 6) and decompensated (n = 7) human RVs. *P < 0.05 decompensated versus control. (D) Representative graph demonstrating the p53 expression in cardiomyocytes (in %) in control (n = 6), compensated (n = 6) and decompensated (n = 7) human RVs. All the data represent the mean ± SEM.

As an increase in apoptotic rate of endothelial cells (ECs) is a characteristic of human decompensated stage hypertrophy [2], we thought to investigate RV p53 expression in compensated versus decompensated stage RVH in humans. p53 was elevated at the decompensated RVH in comparison to controls and individuals with compensated stage hypertrophy on protein level, indicating an involvement of the tumor suppressor p53 in the transition from RV hypertrophy to RV failure (Fig 1A). Similarly, p53 expression was significantly upregulated in ECs of RVs from human hearts with decompensated RVH, as indicated by an increase in the number of CD31-positive cells in conjunction with p53 expression (Fig 1B and 1C). The expression of p53 was unchanged in cardiomyocytes (CMs) between compensated and decompensated RVs in humans (Figs 1D and S1A). Together, our data on human RVs indicate that EC p53 may contribute to EC loss and capillary rarefaction of the cardiac microvasculature, a key hallmark of human maladaptive RV hypertrophy [2].

### Effect of Quinacrine on right heart function in pulmonary arterial banding induced right ventricular hypertrophy

**Fig 2:**
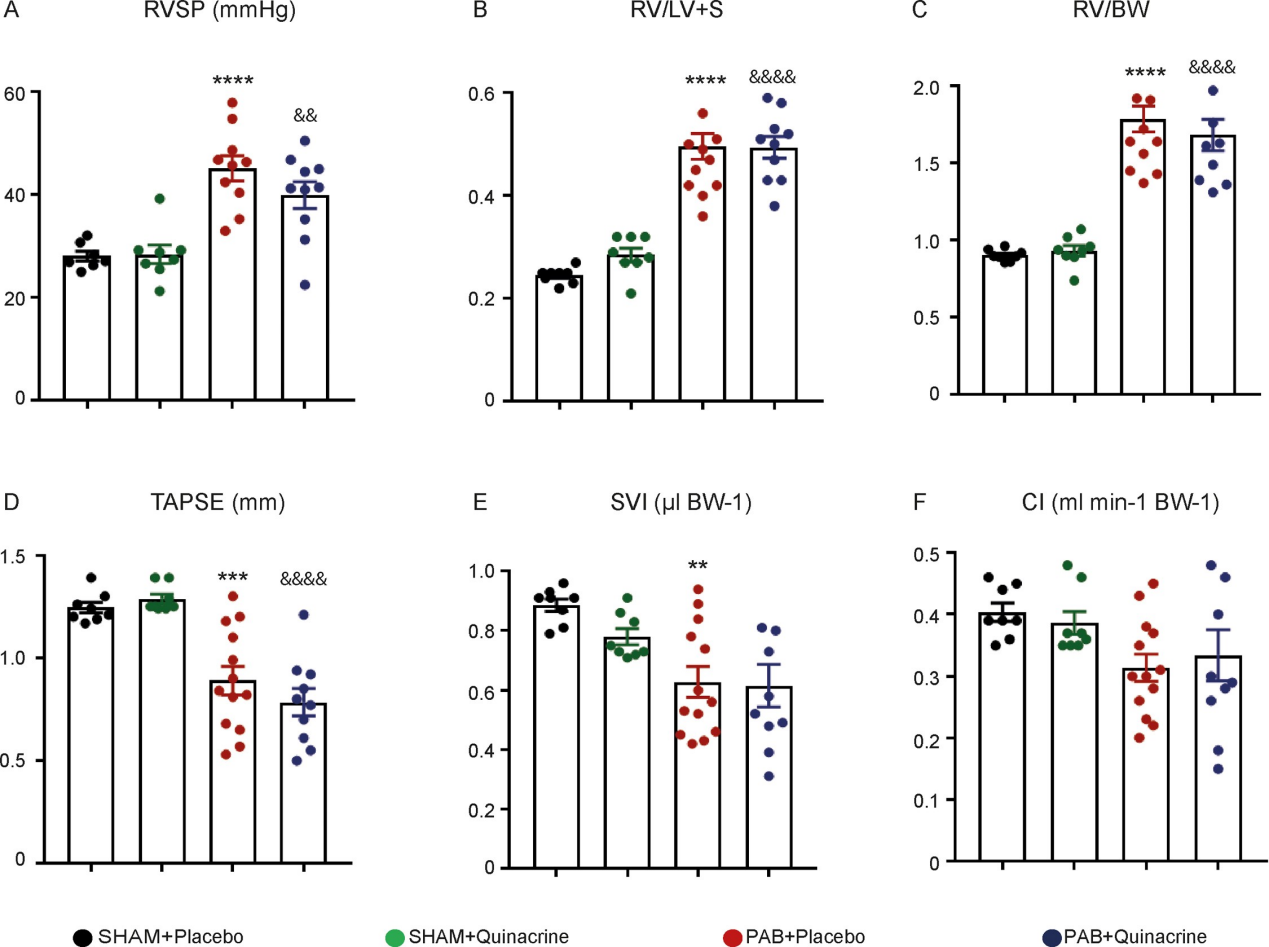
Effect of Quinacrine treatment on right ventricular function in Pulmonary Arterial Banding (PAB) model. (A) Right ventricular systolic pressure (RVSP, mmHg). (B) RV hypertrophy (Fulton index) measured as ratio of RV to LV plus septum (RV/LV+S), (C) Ratio of RV mass to body weight (RV/BW), (D) Tricuspid annular plane systolic excursion (TAPSE, mm), (E) Stroke volume Index (SVI, μl/BW) and (F) Cardiac Index (CI, ml/min/BW) were measured in SHAM (n = 7–8), PAB Placebo (n = 10–13) and PAB mice treated with Quinacrine (n = 10). ****P < 0.0001, **P < 0.01 PAB versus SHAM; ^&&&&^P < 0.0001, ^&&^P < 0.01 PAB+Quinacrine versus SHAM+Quinacrine Data represent the mean ± SEM.

The expression of p53 protein was markedly enhanced three weeks after surgery, in comparison to mice after one week of PAB- and SHAM-operated animals (S1B and S1C Fig). The clipping of the pulmonary artery resulted in a significant increase of RV systolic pressure (RVSP) in all PAB-operated mice in comparison to SHAM-operated mice three weeks after the banding (Fig 2A). No change in RVSP was noted between Quinacrine and Placebo-treated animals. PAB resulted in RV hypertrophy, as indicated by an increase of the Fulton index, the ratio of RV to Left Ventricle plus Septum mass (RV/LV+S) (Fig 2B) and the ratio of RV weight to body weight (RV/BW), in comparison to SHAM-operated mice (Fig 2C). Both RV/LV+S and RV/BW ratios were not affected by Quinacrine treatment (Fig 2B and 2C). The analysis of cardiac function by echocardiography indicated that one week of PAB resulted in worsening of RV function, as determined by significant decrease of tricuspid annular plane systolic excursion (TAPSE) and Stroke Volume Index (SVI) (Fig 2D and 2E), and strong though not significant decline of Cardiac Index (CI) (Fig 2F). Quinacrine treatment did not alter any of the parameters, in both SHAM and PAB-operated groups of mice (Fig 2D and 2F), as well as systemic arterial pressure (SBPsys) (S2A Fig).

### Effect of p53 activation by Quinacrine on RV remodeling in pulmonary arterial banding

**Fig 3:**
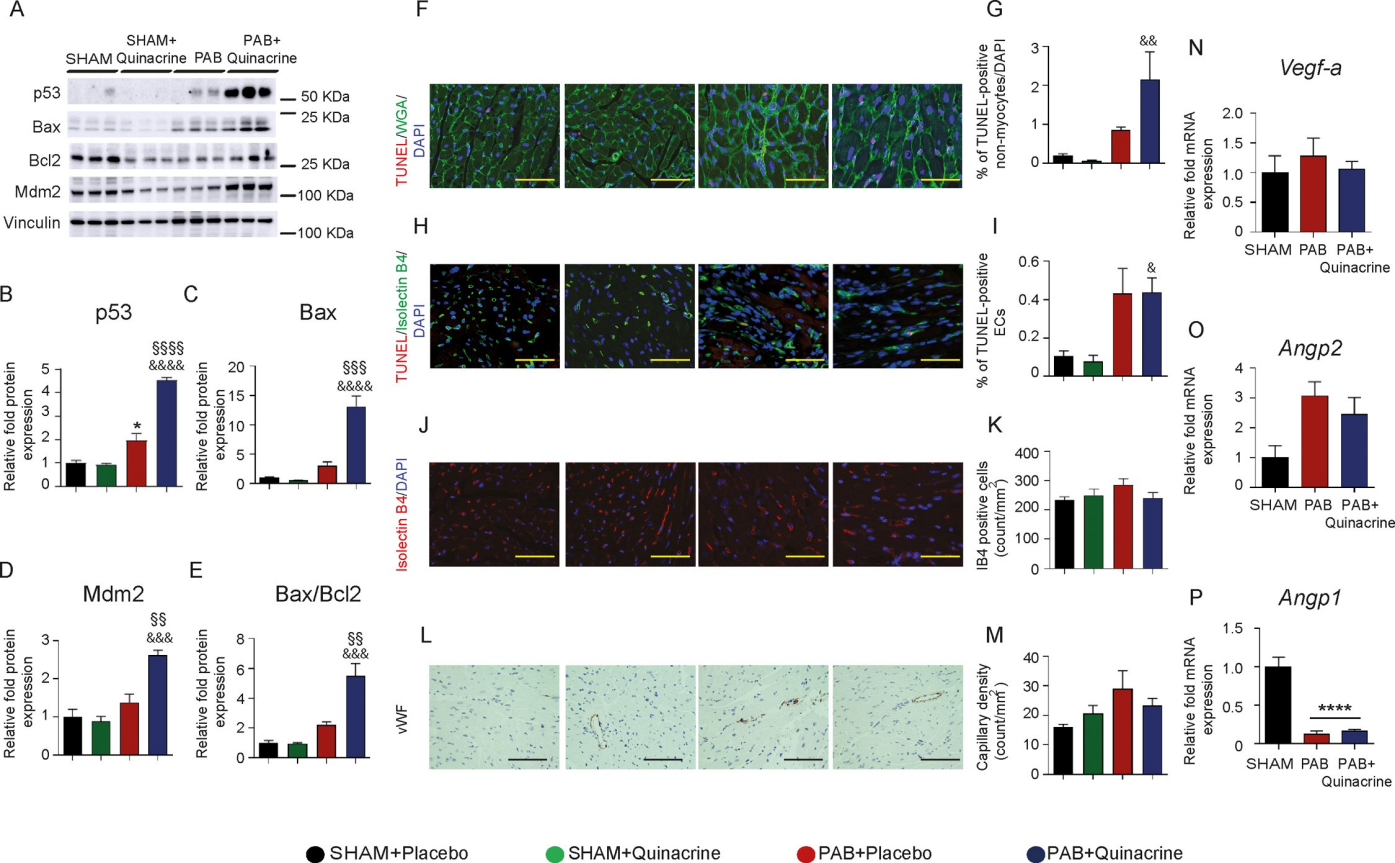
Analysis of p53 activation with Quinacrine treatment on pro-apoptotic and pro-angiogenic signaling. (A) Representative western blot analysis of p53 expression and its downstream targets Bax, Bcl2 and Mdm2 in SHAM, PAB and PAB+Quinacrine- treated mice. (B) Relative fold protein expression of p53 (C) Bax (D) Mdm2 and (E) Bax/Bcl2 ratio. Vinculin served as a loading control. (F) Terminal deoxynucleotidyl transferase dUTP nick end labeling (TUNEL) (in red) shows a significant increase of apoptotic non-myocytes. FITC-labelled wheat germ agglutinin WGA (green) marks cell borders. (G) Representative graph demonstrating an increase in the percentage of TUNEL-positive non-myocytes. (H) TUNEL positive staining on endothelial cells (TUNEL/IB4) (I) Representative graph demonstrating the percentage of TUNEL-positive ECs (J) Endothelial cell number was analyzed by fluorescence labelled Isolectin B4 (IB4) staining. (K) Representative graph for IB4 positive cells per mm^2^ (L) Number of capillaries were analyzed by von Willebrand factor (vWF) staining. (M) Representative graph for capillary density per mm^2^. Scale bars indicates 50 μM. (N) Relative fold mRNA expression of *Vegf-a* (O) *Angp2* and (P) *Angp1* expression in SHAM, PAB and PAB+Quinacrine treated animals (n = 3–5 per group). ****P < 0.0001, *P < 0.05 PAB versus SHAM; ^§§§§^P < 0.0001, ^§§§^P < 0.001, ^§§^P < 0.01, PAB+Quinacrine versus PAB; ^&&&&^P < 0.0001, ^&&&^P < 0.001, ^&&^P < 0.01, ^&^P <0.05, PAB+Quinacrine versus SHAM+Quinacrine. Data represent the mean ± SEM.

Quinacrine treatment resulted in a significant accumulation of p53 protein in the RVs (Fig 3A and 3B), while its expression was not significantly altered in LVsand lungs (S2B and S2C Fig). p53 stabilization by Quinacrine led to an upregulation of its downstream targets Bcl-2-associated X protein (Bax), B-cell lymphoma 2 (Bcl2) and mouse double minute 2 homolog (Mdm2) (Fig 3A and 3C and 3D), as determined by western blot analyses of proteins isolated from RV tissues. Additionally, the ratio of Bax/Bcl-2 was augmented (Fig 3E). Quinacrine administration did not affect the phosphorylation of p65 subunit of nuclear factor NF-kappa-B (NF-κB) and the expression of its downstream transcriptional target, Intercellular Adhesion Molecule 1 (*ICAM1*) (S2D, S2E and S2F Fig). To assess whether p53 accumulation might contribute to RV cardiac cell apoptosis, the TdT-mediated dUTP-biotin nick end labeling (TUNEL) assay was performed in combination with wheat germ agglutinin (WGA) labelling. p53 activation resulted in a prominent increase in TUNEL-positive non-myocytes in Quinacrine-treated animals, as compared to vehicle-treated control mice (Fig 3F and 3G), while no obvious signs of CM cell death were detected. To visualize apoptotic cells in the cardiac vasculature, an endothelial cell marker, fluorescence conjugated *Griffonia simplifolia* Isolectin IB4 (GS-IB4) was applied concomitantly with TUNEL on RV tissue sections. PAB resulted in an induction of TUNEL-positive ECs in comparison to SHAM-operated animals. Quinacrine administration did not further increase a number of TUNEL-positive ECs (Fig 3H and 3I). Concomitantly with these observations, p53 activation neither diminished EC number (IB4 staining) (Fig 3J and 3K), nor the capillary density (von-Willebrand-Factor, vWF staining) (Fig 3L and 3M) on RV tissues 7 days after PAB. The expression of downstream targets of HIF-1α, namely Vascular Endothelial Growth Factor *(VEGF-A)* and Angiopoietin 2 *(Angp2)*, known to promote neo-vascularization in conjunction with each other [23] was not affected neither after PAB, nor after Quinacrine administration (Fig 3N and 3O). The expression of Angiopoietin 1 *(Angp1)* decreased in RVs of Placebo-treated PAB mice, while p53 stabilization did not alleviate this decline (Fig 3P). Similarly, exposure of cardiac ECs to Quinacrine *in vitro* did not affect HIF-1α or VEGF-A expression (S3A–S3D Fig), indicating that Quinacrine-induced p53 activation, does not directly impact HIF-1α-dependent angiogenic gene reprograming in cardiac ECs. Taken together our data indicate, that p53 activation does not induce detrimental effects on RV angiogenesis one week after the PAB.

### Effect of p53 activation on markers of sarcomere assembly, cardiac contractility, glycolysis and mitochondrial function

**Fig 4:**
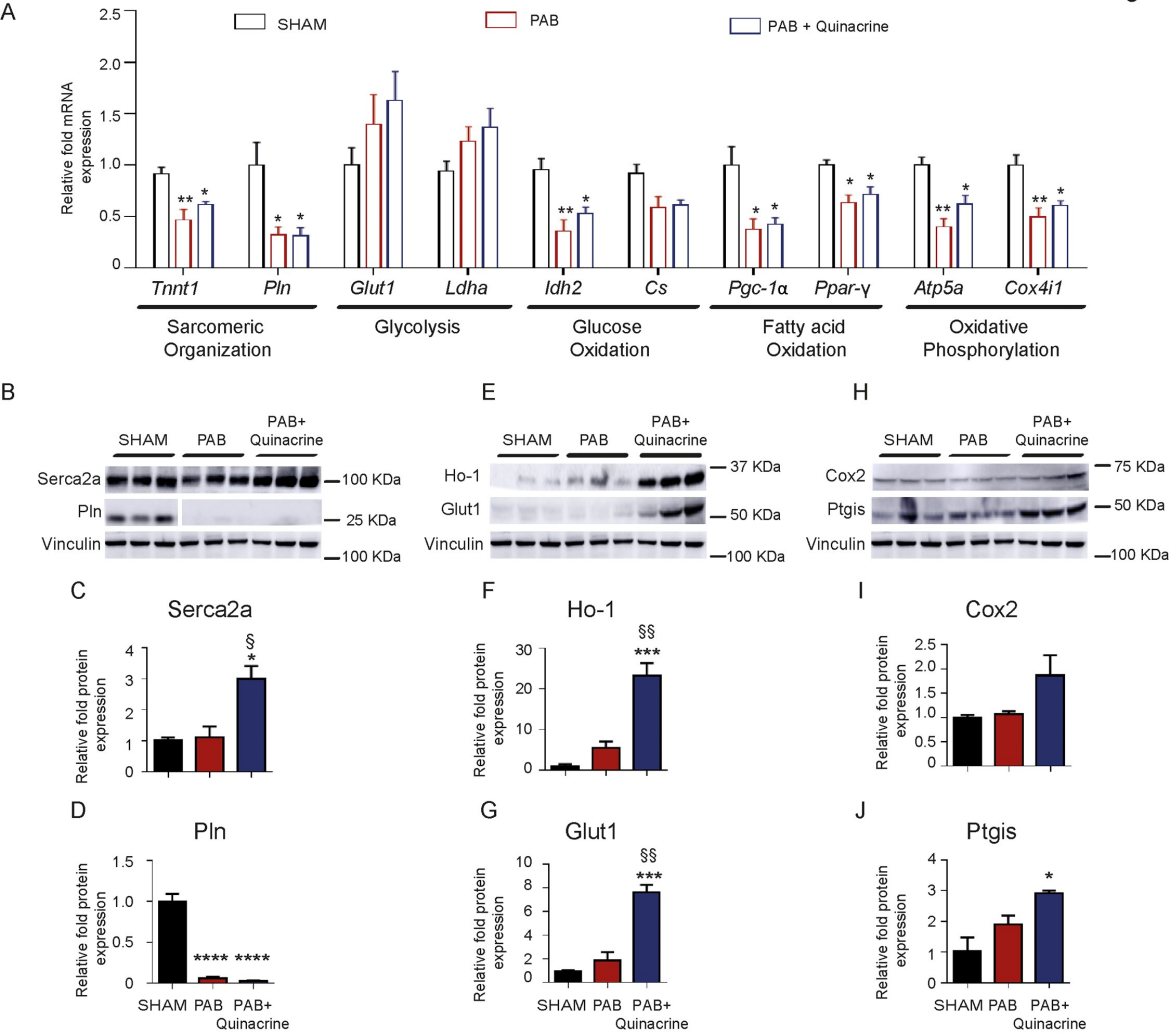
Effect of p53 activation with Quinacrine treatment on markers of sarcomere organization, glycolysis, fatty acid and mitochondrial metabolism and respiration. (A) Relative fold mRNA expression of Troponin T1 (*Tnnt1*), Phospholamban (*Pln*), Glucose Transporter 1 (*Glut1*), Lactate Dehydrogenase A (*Ldha*), Isocitrate dehydrogenase (*Idh2*), Citrate Synthase (*Cs*), peroxisome proliferator-activated receptor gamma coactivator 1-alpha (*Pgc-1α*), peroxisome proliferator-activated receptor gamma (*Ppar-γ*), ATP Synthase F1 Subunit Alpha (*Atp5a*) and Cytochrome C Oxidase Subunit 4 isoform (*Cox4i*) in SHAM (n = 4), PAB (n = 4), and PAB+Quinacrine (n = 5) mice. (B) Representative western blot analysis and (C, D) subsequent densitometric quantification of Sarco/endo plasmic reticulum calcium (Ca2+) ATPase cardiac isoform (Serca2α) and Pln expression in RV tissues of SHAM, PAB and PAB+Quinacrine treated mice (n = 3 per group). (E) Representative western blot analysis and (F, G) subsequent densitometric quantification of Ho-1 and Glut1 in RV tissues of SHAM, PAB and PAB+Quinacrine treated mice (n = 3 per group). (H) Representative western blot analysis and (I, J) subsequent densitometric quantification of Cyclooxygenase 2 (Cox2) and Prostaglandin I2 Synthase (Ptgis) in RV tissues of SHAM, PAB and PAB+Quinacrine treated mice (n = 3 per group). Vinculin served as a loading control. ****P < 0.0001, ***P < 0.001, **P < 0.01, *P < 0.05 versus SHAM; ^§§^P < 0.01, ^§^P < 0.05 PAB+Quinacrine versus PAB. Data represent the mean ± SEM.

PAB resulted in a marked and significant decline of canonical markers of sarcomere disassembly and cardiac contractility, Troponin T1 (*Tnnt1*) and Phospholamban (*Pln*) (Fig 4A). No significant changes in the regulation of these transcripts have been noted upon p53 activation (Fig 4A). Sarco/endoplasmic reticulum Ca^2+^-ATPase (Serca2α)/Phospholamban (Pln) interactome regulates the changes in Ca(2+)-ATPase (Serca2α) activity, which represent a fundamental determinant of cardiac contractility [24–27]. Remarkable diminished Pln expression was noted in the RVs of PAB operated mice in comparison to the RVs of SHAM-operated mice (Fig 4B and 4D). Interestingly, Quinacrine administration enhanced Serca2α accumulation (Fig 4B and 4C). The myocardium of compensated RV is characterized by increased levels of Glucose Transporter 1 (*Glut1*), promoting increased glucose uptake [13]. Marked, though not significant elevation in mRNA expression of key markers of enhanced glycolysis, Glucose Transporter Type 1 (*Glut1*) and Lactate Dehydrogenase A (*Ldha*) (Fig 4A) in RVs of PAB mice confirms previous results obtained from the banded rats [28]. Interestingly, Quinacrine administration further promoted Glut1 protein expression (Fig 4E and 4G). PAB diminished the expression of mRNA transcripts encoding enzymes involved in aerobic glucose catabolism, Isocitrate Dehydrogenase 2 (*Idh2*) and Citrate Synthase (*Cs*) while Quinacrine administration did not change the expression of those enzymes (Fig 4A). Peroxisome proliferator-activated receptor gamma coactivator 1-alpha (PGC-1α) regulates mitochondrial biogenesis along with oxidative metabolism [29]. As expected, *Pgc-1α* transcripts declined in RVs of banded mice (Fig 4A). A reduction of Pgc-1α was concomitant to the reduction of peroxisome proliferator-activated receptors gamma (*Ppar-γ)*, a subject to transcriptional co-activation by Pgc-1α (Fig 4A*)*, while p53 activation did not further modulate their decay (Fig 4A). Lastly, we looked for the changes in mRNA expression of key components of mitochondrial respiratory chain complexes. There was a marked decline of the transcripts of Cytochrome c Oxidase Subunit 4 Isoform 1 (*Cox4i1*) and ATP synthase F1 subunit alpha (*Atp51a*), of the enzyme catalyzing ATP synthesis during mitochondrial phosphorylation (Complex V) in RV tissues of PAB mice (Fig 4A). Again, p53 activation, driven by Quinacrine, did not further diminish the transcript levels of the oxidative phosphorylation machinery (Fig 4A). The expression of heme oxygenase 1 (HO-1), critically involved in prevention of vascular inflammation, was induced in RVs of mice treated with Quinacrine (Fig 4E and 4F). Furthermore, the expression of key enzymes of prostanglandin processing, Cyclooxygenase 2 (Cox2), and Prostacyclin Synthase (Ptgis), responsible for conversion of prostaglandin H2 to prostacyclin (prostaglandin I2) were not significantly altered in RVs of PAB-operated mice in comparison to RVs of SHAM-operated control mice, though p53 accumulation augmented Cox2 and Ptgis protein accumulation (Fig 4H, 4I and 4J). Taken together our data indicate that p53 activation induced by Quinacrine does not exert major impact on markers of sarcomere organization, fatty acid metabolism, mitochondrial metabolism and respiration, but modulated the expression of several anti-inflammatory and vaso-protective proteins.

### Effect of p53 activation on gene expression in cardiomyocytes and cardiac endothelial cells

**Fig 5:**
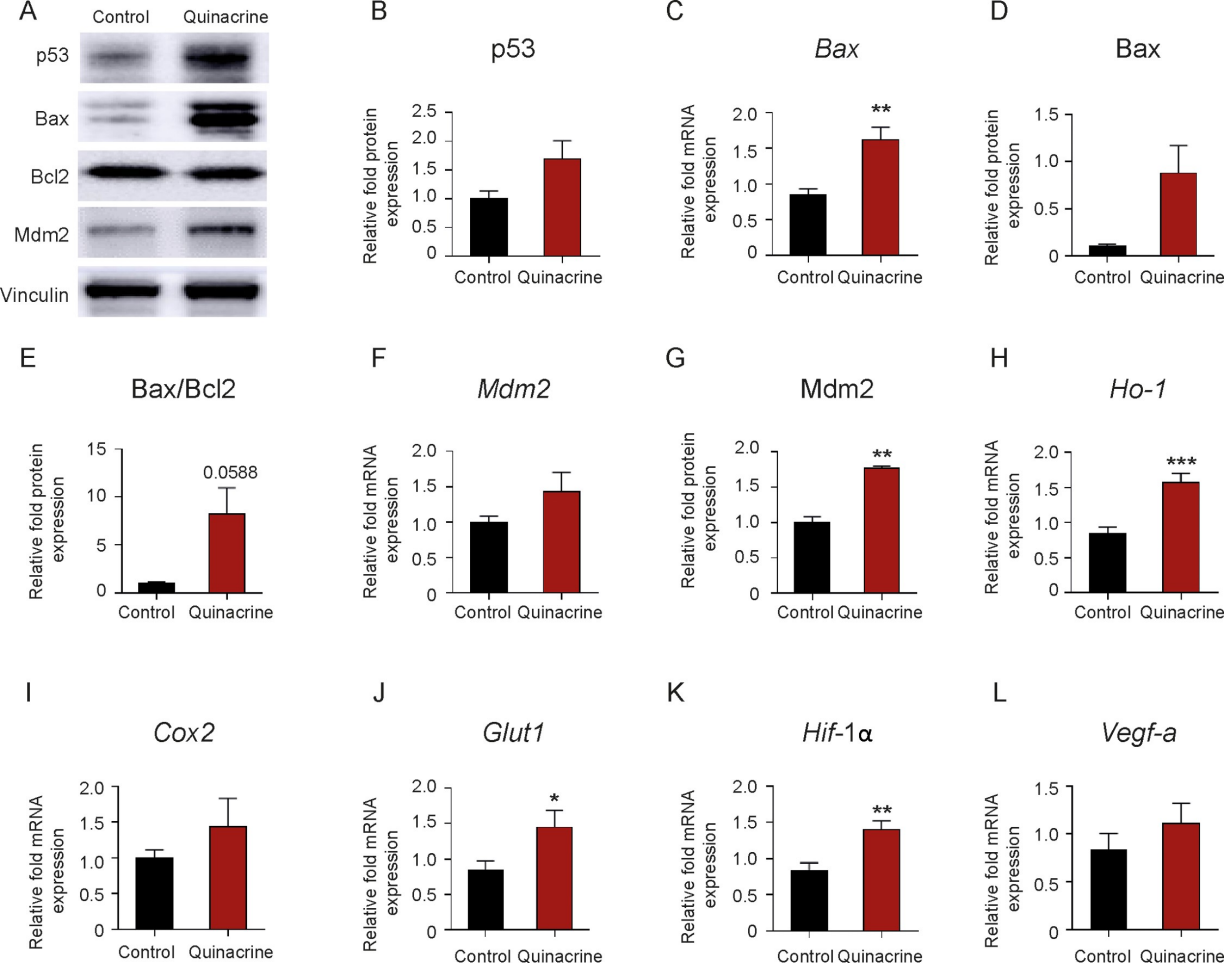
Effect of p53 activation with Quinacrine treatment on RV adult rat cardiomyocytes. (A,B) Representative western blot analysis and subsequent densitometric quantification of p53, (D) Bax, (E) Bax/Bcl2 and (G) Mdm2 in adult RV cardiomyocytes (CMs) after Quinacrine administration, in comparison to DMSO-treated control cells. Vinculin served as a loading control. (E) Relative fold change mRNA expression of (C) *Bax*, (F) *Mdm2*, (H) *Ho-1*, (I) *Cox2*, (J) *Glut1*, (K) *Hif-1α* and (L) *Vegf-a* in RV adult cardiomyocytes upon Quinacrine treatment, normalized to *beta-actin* (n = 7 per group) ***P < 0.001. **P < 0.01, *P < 0.05 for Quinacrine treatment versus DMSO control. Data represent the mean ± SEM.

To substantiate the *in vivo* results, adult rat RV CMs were exposed to Quinacrine. Quinacrine exposure resulted in p53 accumulation (Fig 5A and 5B), an increase of Bax (Fig 5C and 5D), Bax/Bcl2 ratio (Fig 5E), and Mdm2 protein levels (Fig 5F and 5G) confirming activation of the p53 pathway. Alongside, Quinacrine treatment of CMs induced the expression of genes, like *Ho-1*, *Cox2* and *Glut1* (Fig 5H–5J). Although Quinacrine exposure of RV CMs resulted in a strong increase of Hif-1α and *Vegf-a* expression (Fig 5K and 5L), on human cardiac microvascular endothelial cells (hMVECs) no effect on HIF-1α and VEGF-A expression was noted (see S3A–S3D Fig).

### Effect of p53 activation on RV remodeling and fibrosis

**Fig 6:**
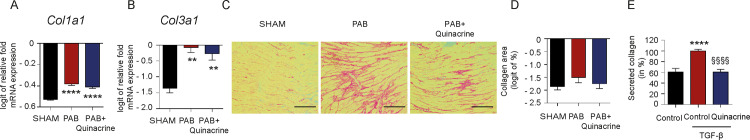
Effect of p53 activation with Quinacrine treatment on fibrosis in PAB treated RV tissue and adult mouse cardiac fibroblasts. Relative fold mRNA expression of (A) Collagen 1a1 (*Col1a1*) and (B) Collagen3a1 (*Col3a1)* in SHAM (n = 4), PAB (n = 4) and PAB+Quinacrine (n = 5) RV tissues. ****P < 0.0001, **P < 0.01 versus SHAM (C) Sirius red staining representing collagen expression in SHAM (n = 5), PAB (n = 5) and PAB+Quinacrine (n = 5) RV sections. Scale bars indicates 50 μM. (D) Representative graph indicating total collagen area (logit of %) in all three studied groups. (E) Secreted collagen (in %) from cardiac fibroblasts in control (DMSO), TGF-β and TGF-β+Quinacrine (3μM) (n = 3). Data represent the mean ± SEM.

Next, we examined the impact of p53 activation on the degree of fibrosis in the PAB model of RVH. One week of PAB resulted in elevated expression of *Col1a1* and *Col3a1* transcripts (Fig 6A and 6B). A marked though not significant initiation of fibrotic response, determined by sirius red staining, was detected in RVs of Placebo-treated animals in comparison to RVs of SHAM-operated control mice (Fig 6C and 6D). Quinacrine administration did not further promote fibrosis induction (Fig 6A–6D). Interestingly, the exposure of murine cardiac fibroblasts to Quinacrine diminished Transforming growth factor beta TGF-β1 induced collagen synthesis *in vitro* (Fig 6E), indicating that p53 might play a protective role on RV fibrosis rate at the later stages of RVH.

## Discussion

The tumor suppressor p53 is known to be activated in response to cellular stress, e.g. hypoxia and induces apoptosis in various cell types [30]. In several heart diseases associated with oxidative and mechanical stress, p53 related apoptosis has been reported [8, 31–33]. In addition to its cell death-inducing role, p53 has the potential to increase cell survival pathways [34, 35]. In the present study, we determined the effect of p53 activation by Quinacrine on right ventricular remodeling and function in a pulmonary artery banding (PAB) model of RV hypertrophy one week after the surgery, the time point, at which p53 levels were negligibly different to SHAM-operated controls. Although, Quinacrine treatment at the compensated stage of RVH resulted in a strong p53 accumulation with activation of its downstream signaling, both RV hypertrophy and RV function have not been significantly impaired.

The p53 accumulation inhibits HIF-1α dependent angiogenesis, resulting in an impairment of left ventricular function and emergence of maladaptive remodeling at the compensated stage [8]. However, in another report, no evidence of alterations in HIF-1α and capillary density was noted in the model of compensated LVH, which does not further progress to LV failure [36]. HIF-1α via binding to Mdm2, blocks the degradation of p53 and inhibits Mdm2-mediated nuclear export of p53 [37]. p53 has been shown to be elevated only in severe hypoxia or sustained pressure overload, while HIF-1α accumulates both in mild and severe hypoxia [8, 38]. In cancer cells, p53 impairs HIF-1α transcriptional activity, while high p53 level causes HIF-1α degradation [39]. Our data show that p53 accumulation induced by Quinacrine did not alter the HIF-1α expression and its downstream targets of the angiogenic gene program. Furthermore, our *in vitro* experiments in cardiac ECs, indicated that p53 activation by Quinacrine had no impact on HIF-1α transcription and HIF-1α protein accumulation. Consequently, our studies reveal that p53 stabilization did not impair cardiac angiogenesis, defined by maintained number of vWF-positive vessels and cardiac ECs and the steady state expression of several transcripts of HIF-1α angiogenic gene program. Concomitantly to this, the exposure of RV CMs to Quinacrine maintained HIF-1α angiogenic gene program. Thus, our results are in agreement with some of the previous reports from the compensated LVH [36]. Despite of p53 accumulation and in contrast to the data from pressure-overloaded LVH, in which Quinacrine significantly increased CM and EC apoptosis at compensated stage [8], p53 accumulation in the RVs in our case had no effect on CM apoptosis. Regardless of augmentation of TUNEL-positive cardiac cells upon p53 accumulation, both EC apoptosis and vascular remodeling were not significantly altered.

Concomitantly with accumulated p53, strong upregulation of stress response anti-oxidant Heme Oxygenase 1 (HO-1), an essential cardio-protective and anti-apoptotic factor [40–43], responsible for reduction in oxidative and inflammatory insults [41, 44], preservation of RV microcirculation, and maintenance of RV function [3] was noted.

While the levels of Phospholamban (Pln), a key regulator of sarco/endoplasmic reticulum Ca^2+^-ATPase pump activity in the heart has been massively reduced both in the Placebo- and Quinacrine-treated PAB mice, p53 accumulation markedly elevated Serca2α protein expression. Enhancing Serca activity has been demonstrated to improve the contractile function in failing human cardiomyocytes and in acute Ca^2+^-overload-induced LV dysfunction in rat hearts [45]. Interestingly, Quinacrine was shown to protect adult cardiomyocytes from the injury, by delaying of cell death of both metabolically inhibited and ischemic cells [46] and by means of enhancement of improved cardiomyocyte calcium signaling [47]. Although the upregulation of both (HO-1 and Serca2α) cardio-protective factors has been noted in Quinacrine treated PAB mice, yet no relevant functional outcomes of their increase were investigated. Furthermore, this study did not evaluate the expression profile of HO-1 and Serca2α in SHAM-operated Quinacrine treated mice. Interestingly, Quinacrine-treated PAB mice show markedly enhanced levels of Mdm2. Mdm2 interacts with p53 and has been shown to inhibit p53 transcriptional activity [48]. Thus an increase of Mdm2 in Quinacrine-treated PAB mice may repress the p53 transactivation potency toward HIF-1α and counterbalance HIF-1α related angiogenesis. On the other side, an increase of Mdm2 at the initial stages after PAB might speak for the potential contribution of Mdm2 ligase to cardiomyocyte survival, which has been already proposed for ischemia-reperfusion model of cardiac cell death, in which cardiomyocytes become less susceptible to cell death [49]. An induction of p53 has been linked to the inhibition of NF-κB, while down-regulation of p65 phosphorylation suggested to serve as the primary mechanism of Quinacrine action [18]. Interestingly, the p53 activation did not alter neither the phosphorylation of p65 subunit of NF-κB, nor the expression of ICAM1 and Cox2, indicating that Quinacrine did not impair NF-κB mediated signaling and transcription after PAB.

Recent evidence suggests that p53 affects cardiac architecture, contractility, mitochondrial biogenesis, glycolysis and oxidative phosphorylation capacity of adult CMs, signifying p53 as a master regulator of the cardiac transcriptome [17]. Moreover, p53 has been established to induce transcriptional activation of various genes, critical regulators of glycolysis, as glucose transporters, Glut1/4, Hexokinase 2 (Hk2) and Phosphoglycerate mutase (Pgm) [50]. Keeping this in mind, we sought to investigate the profile of genes involved in excitation-contraction coupling, energy metabolism, and the oxidative stress response after p53 activation upon PAB. Important to note that, similarly with the previous data in PAB rats [28], our banded mice exhibited elevation of transcripts of genes encoding factors necessary for glycolysis, as Glut 1 and Ldha, whereas Quinacrine further promoted Glut1 protein upregulation. Glut1 has been proposed to play a protective role at compensated stage of RVH in the MCT model of PAH [13]. Concomitantly with dysregulation in the expression profile of key markers of glycolysis (Glut1, LDHA), PAB triggered decline of the genes encoding key rate-limiting enzymes for aerobic glucose oxidation (ISD, CS), was not majorly affected by Quinacrine. In rats and in patients with PAH, maladaptive RV hypertrophy is characterized by a significant reduction in the expression of PGC-1α and its corresponding nuclear receptors PPAR-α, PPAR-γ, and ERR-α [28]. The PPAR-γ recognized to suppress cardiac growth and protect the heart from oxidative damage [51–53]. A decline in the expression of PPAR-γ and PCG-1α noted one week after PAB, was not affected by p53 activation. Beyond the dysregulation of critical components of fatty acid metabolism, the major constituents of mitochondrial respiration, as decline in the expression of Atp5a1 and Cox4i, indicative of reduction of ATP generation, were not affected by p53 accumulation.

Oxidative stress may be critical for activation of cell death in the overloaded heart [31] and p53 has been demonstrated to play a dual role for reactive oxygen species (ROS) regulation. On one side, down-regulation of p53 results in elevation of intracellular ROS, indicating for the antioxidant function of the p53 [54]. Thus, p53 in addition to causing tissue injury, drives the expression of genes of antioxidant response, associated with a decrease in intracellular ROS and playing protective role, such as HO-1, which generates the antioxidant, biliverdin [55]. HO-1, recognized as a cyto-protective factor in various tissues [56], has been determined to play an essential role in protecting the CMs from apoptosis [57]. Similarly, Cox2, another p53 downstream target, has been shown to be induced by ROS and oxidants [58, 59]. The induction of Cox2 essential for prostaglandin formation also protect CMs from oxidant injury, representing another illustration of an adaptive response that shields the cells from oxidant stress [59]. Importantly, a rise in mitochondrial ROS (mROS) and accumulation of a redox sensitive transcription factor p53 at maladaptive RV remodeling in MCT model of PAH has been already noted [13]. This finding is correlated with our observation that p53 is accumulated in human RVs of decompensated stage RV hypertrophy and in mouse RVs at the later stages of PAB. Although p53 protein expression enhanced in cardiac ECs of decompensated stage, Quinacrine-induced p53 accumulation did not efficiently promote EC apoptosis early after PAB. Subsequently, an accumulation of tumor suppressor p53 protein did not have deleterious effects on RV function one week after the PAB. A limitation of the current study is that the analyses were performed one week after the PAB, at which pro-survival mechanisms may be actively involved. The p53 activation at the later RVH may enhance pro-apoptotic signaling pathways, which ultimately may result in an impairment of RV function. The mechanisms of p53-driven transcriptome are very complex, and may include many potential cellular targets and Quinacrine may drive potential drug off-target effects. Upregulation of several factors, observed in RVs *ex vivo* and in RV cardiomyocytes *in vitro*, as HO-1, Cox2 and Glut1 may trigger a compensatory adaptations beneficial for the stressed RVs. Whether the differences between RV versus LV microvascular remodeling are responsible for the distinct p53-dependent responses between the two ventricles requires further investigation.

## Supporting information

S1 Fig p53 expression in cardiomyocytes of human RVs and in mouse RVs of PAB model. (A) Representative images of p53 expression in cardiomyocytes of control (n = 6), compensated (n = 6) and decompensated (n = 7) human RVs. Smaller and bigger scale bars indicate 10 and 20μM, respectively. (B) Immunoblot analysis and (C) subsequent densitometric quantification of p53 expression in SHAM-operated (n = 10), PAB-operated (7 days) (n = 7) and PAB-operated (21 days) mice (n = 8). *P < 0.05, 21 days of PAB versus SHAM, ^§^P < 0.05, 21 days of PAB vs 7days of PAB. Data represent the mean ± SEM.
(PDF)

S2 Fig Effect of p53 activation on nuclear factor NF-kappa-B (NF-κB) signaling. (A) Systemic arterial pressure (SBPsys) measured in SHAM (n = 7), SHAM+Quinacrine (n = 8), PAB (n = 10) and PAB+Quinacrine (n = 8) mice. (B, C) Immunoblot analyses of p53 expression in left ventricular (LVs) and lung tissues of SHAM (n = 3), SHAM+Quinacrine (n = 3), PAB (n = 3) and PAB+Quinacrine (n = 3) groups of mice. (D, E) Immunoblot analysis and subsequent densitometric quantification of phospho-p65 subunit (Ser536) of NF-kB in RVs of SHAM (n = 3), SHAM+Quinacrine (n = 3), PAB (n = 3) and PAB+Quinacrine (n = 3) groups of mice. *P < 0.05, SHAM+Quinacrine vs SHAM. (F) Relative fold mRNA expression of Intercellular Adhesion Molecule 1 (*ICAM1*), normalized to **18S rRNA** in RVs of SHAM (n = 4), PAB (n = 4) and PAB+Quinacrine (n = 4) mice. Data represent the mean ± SEM.
(PDF)

S3 Fig Effect of p53 activation in cardiac endothelial cells. (A) Immunoblot analysis and subsequent densitometric quantification of (B) p53, (C) HIF-1α and (D) VEGF-A protein expression in human cardiac microvascular endothelial cells 24 hours after Quinacrine (6 μM) treatment. DMSO-treated cells served as a negative control. **P < 0.01 Quinacrine treatment versus DMSO control. Data represent the mean ± SEM.
(PDF)

S1 Data (DOCX)

S! Table (XLSX)

S1 Raw images (PDF)
