# Supplementary material for: Effect of p53 activation on experimental right ventricular hypertrophy
Source: PLoS One. 2020 Jun 19;15(6):e0234872. doi: 10.1371/journal.pone.0234872 (PMC7304610; doi:10.1371/journal.pone.0234872)
Supplement: S1 Fig — (A) Representative images of p53 expression in cardiomyocytes of control (n = 6), compensated (n = 6) and decompensated (n = 7) human RVs. Smaller and bigger scale bars indicate 10 and 20μM, respectively. (B) Immunoblot analysis and (C) subsequent densitometric quantification of p53 expression in SHAM-operated (n = 10), PAB-operated (7 days) (n = 7) and PAB-operated (21 days) mice (n = 8). *P < 0.05, 21 days of PAB versus SHAM, §P < 0.05, 21 days of PAB vs 7days of PAB. Data represent the mean ± SEM. (PDF) [file pone.0234872.s001.pdf]

S1 Fig

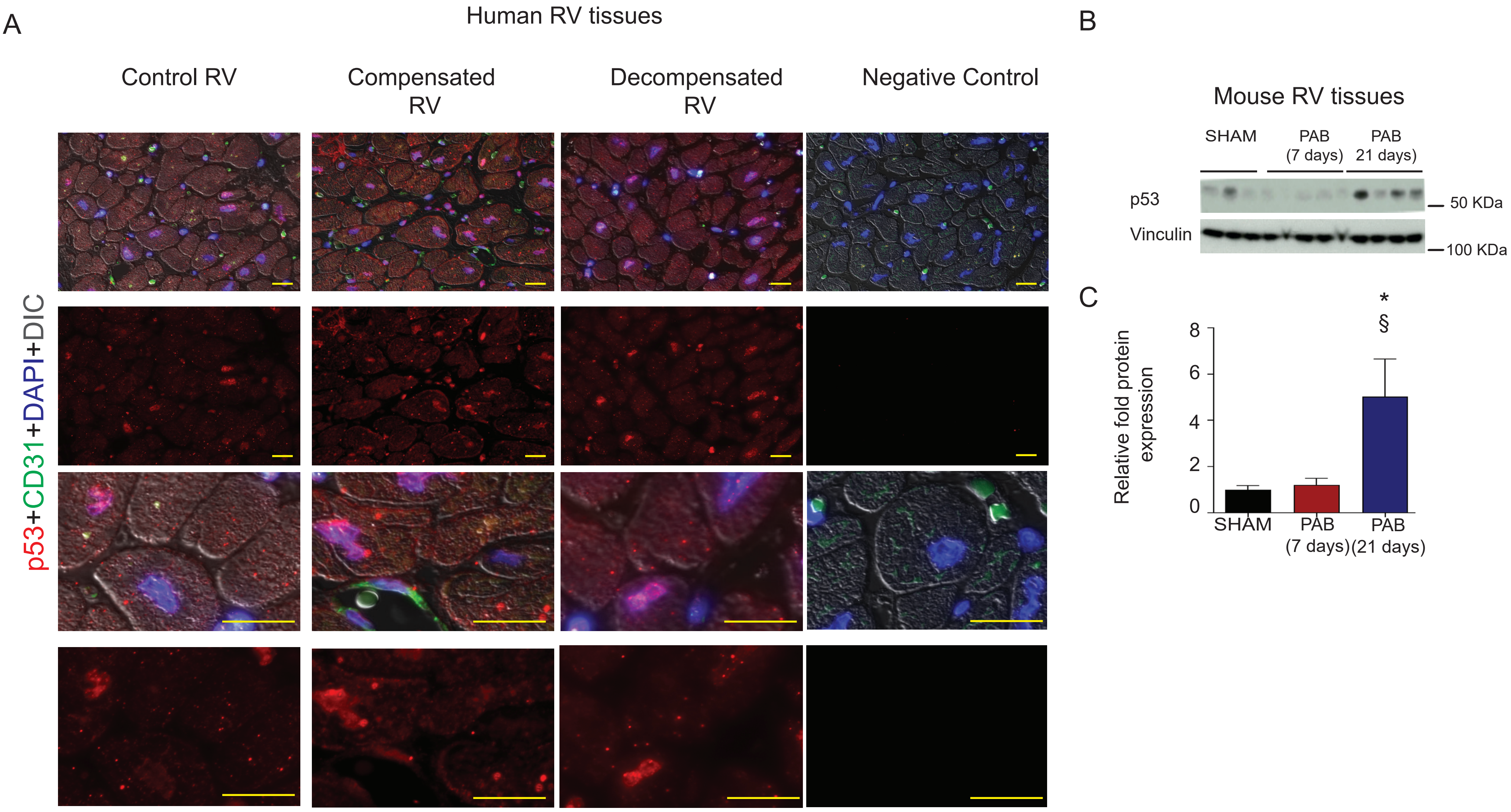
A
Human RV tissues
Control RV
Compensated RV
Decompensated RV
Negative Control
p53+CD31+DAPI+DIC
B
Mouse RV tissues
SHAM
PAB (7 days)
PAB 21 days)
p53
Vinculin
50 KDa
100 KDa
C
Relative fold protein expression
8
6
4
2
0
*
§
SHAM
PAB (7 days)
PAB (21 days)
