# Supplementary figures and images for: Effect of p53 activation on experimental right ventricular hypertrophy
Source: PLoS One. 2020 Jun 19;15(6):e0234872. doi: 10.1371/journal.pone.0234872 (PMC7304610; doi:10.1371/journal.pone.0234872)

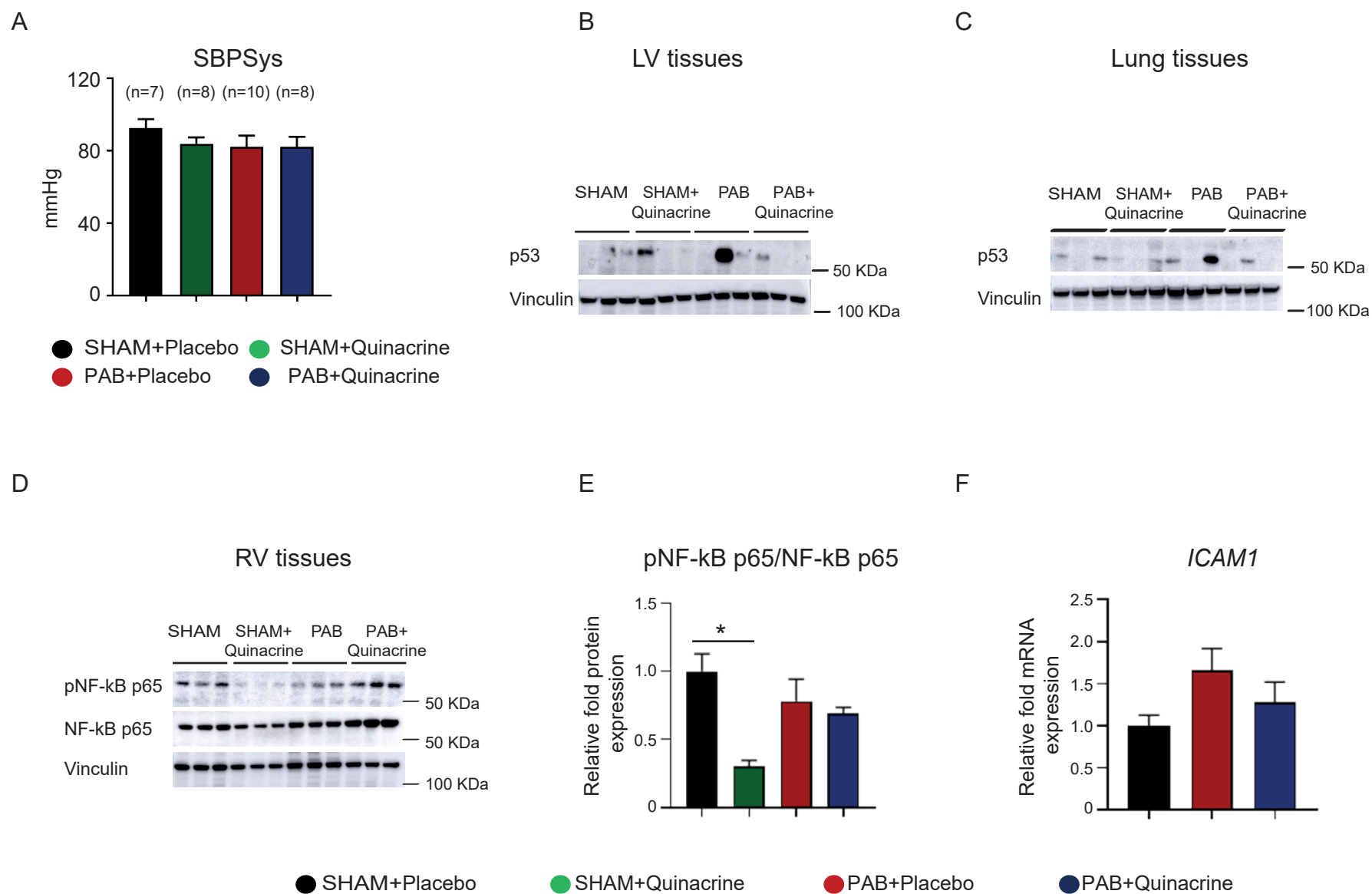

Supplement: S2 Fig — (A) Systemic arterial pressure (SBPsys) measured in SHAM (n = 7), SHAM+Quinacrine (n = 8), PAB (n = 10) and PAB+Quinacrine (n = 8) mice. (B, C) Immunoblot analyses of p53 expression in left ventricular (LVs) and lung tissues of SHAM (n = 3), SHAM+Quinacrine (n = 3), PAB (n = 3) and PAB+Quinacrine (n = 3) groups of mice. (D, E) Immunoblot analysis and subsequent densitometric quantification of phospho-p65 subunit (Ser536) of NF-kB in RVs of SHAM (n = 3), SHAM+Quinacrine (n = 3), PAB (n = 3) and PAB+Quinacrine (n = 3) groups of mice. *P < 0.05, SHAM+Quinacrine vs SHAM. (F) Relative fold mRNA expression of Intercellular Adhesion Molecule 1 (ICAM1), normalized to 18S rRNA in RVs of SHAM (n = 4), PAB (n = 4) and PAB+Quinacrine (n = 4) mice. Data represent the mean ± SEM. (PDF) [file pone.0234872.s002.pdf]

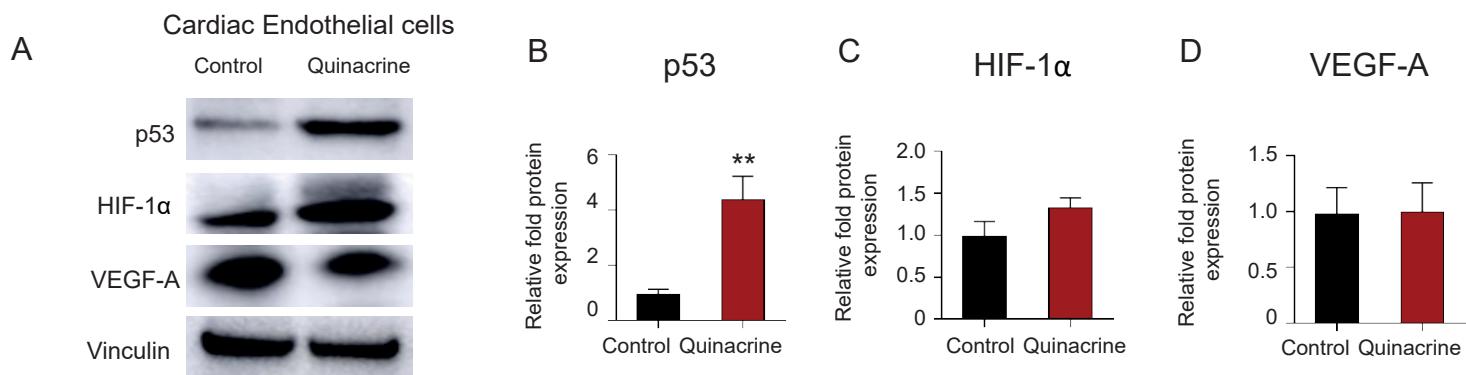

Supplement: S3 Fig — (A) Immunoblot analysis and subsequent densitometric quantification of (B) p53, (C) HIF-1α and (D) VEGF-A protein expression in human cardiac microvascular endothelial cells 24 hours after Quinacrine (6 μM) treatment. DMSO-treated cells served as a negative control. **P < 0.01 Quinacrine treatment versus DMSO control. Data represent the mean ± SEM. (PDF) [file pone.0234872.s003.pdf]
