# Supplementary material for: Effect of p53 activation on experimental right ventricular hypertrophy
Source: PLoS One. 2020 Jun 19;15(6):e0234872. doi: 10.1371/journal.pone.0234872 (PMC7304610; doi:10.1371/journal.pone.0234872)
Supplement: S1 Data — (DOCX) [file pone.0234872.s004.docx]

**Supplementary data**

**Cell culture**

**Isolation of adult rat ventricular cardiomyocytes**

Ventricular heart muscle cells were isolated from rats as described in greater detail previously [1]. Briefly, hearts were excised under deep anaesthesia, transferred rapidly to ice-cold saline, and mounted on the cannula of a Langendorff perfusion system. Hearts were perfused first for 10 minutes in a non-re-circulating manner with a calcium-free perfusion buffer, then for 20–25 minutes in a re-circulating manner in a buffer supplemented with collagenase and 25µmol/l calcium. Thereafter, RV and LV were separated and RVs were minced separately and incubated for another 5 minutes in re-circulating buffer. The remaining cell solution was filtered through a 200µm nylon mesh. The suspension was centrifuged at 25xg for 10 minutes to pellet down cardiomyocytes (CM), while the supernatant contained mostly the endothelial cells (ECs) and fibroblasts. CMs were re-suspended in buffer with a stepwise increase in calcium and finally transferred to M199 culture medium [supplemented with carnitine (2mmol/l), creatine (5mmol/l), and taurine (5mmol/l)]. Rat cardiomyocytes were attached to culture dishes by pre-coating of the dishes with 4% (vol/vol) foetal calf serum (FCS). One hour after plating, cell culture medium was changed and CMs were treated with 3 µM of Quinacrine for 24-30 hours and processed for RNA or protein isolation, respectively.

**Isolation of adult mouse ventricular cardiac fibroblasts**

Adult murine cardiac fibroblasts (CFs) were isolated as previously described [2]. After 24 hours of starvation in the fibroblast medium (Cell Applications, San Diego, USA), CFs were stimulated with 10ng/ml Transforming growth factor beta (TGF-β) and 3 µM of Quinacrine for 72 hours. Dimethyl sulfoxide (DMSO) was used as a negative control.

**Culturing of human cardiac microvascular endothelial cells**

Human cardiac microvascular endothelial cells (hMVECs) were purchased from Lonza (CC-7030, Basel, Switzerland) and cultured as per manufacturer’s instructions. Human HMVECs split into 6 cm dishes were treated with 6µM of Quinacrine or DMSO, which served as a control. After 24 hours, protein lysates were collected in RIPA cell lysis buffer (Thermo Fisher Scientific, Massachusetts, USA).

**Collagen synthesis in cardiac fibroblasts**

Cardiac fibroblasts (CFs) were cultured in serum-free media for 24 hours. Next day, cells were treated with 3μM Quinacrine followed by TGF-β1 (10 ng/ml) stimulation for another 72 hours. L-Ascorbic acid (0.25 mM) was added every day to the medium. After 72 hours of treatment, media was collected and total collagen content was assessed by Sircol soluble collagen assay kit (Biocolor, Carrickfergus, County Antrim, United Kingdom) as per manufacturer’s instructions.

**Western blot analysis**

Total protein extracts were isolated from RV tissues with Cell lysis buffer containing Halt Protease and Phosphatase Inhibitor Cocktail mix and homogenized with Tissue Lyser LT (Qiagen, Hilden, Germany). Homogenates were centrifuged at 12000 rpm for 15 min at 4°C. Protein concentration was measured using Bio-Rad DC Protein Assay (Bio-Rad Laboratories, California, USA) and normalized to equal concentrations with NuPAGE LDS Sample Buffer and NuPAGE Sample Reducing Agent. 30-50µg of proteins were separated on NuPage 4-12% Bis-Tris Gels (Thermo Fisher Scientific, Massachusetts, USA) and blotted on nitrocellulose membranes (Bio-Rad Laboratories, California, USA). Membranes were blocked in 5% milk in Tris-Buffered Saline with addition of Tween 20 (TBS/T) for 1 hour. All primary antibodies were diluted at 1:500 concentration in 5% BSA in TBS/T and incubated for overnight at 4°C followed by incubation with HRP conjugated secondary antibodies (GE Healthcare, Illinois, USA) were diluted in 5% milk in TBS/T. Membranes were developed with Amersham ECL prime western blot analyses detection reagent in Amersham Imager 600 and signal intensity was quantified using Amersham Imager 600 software.

**Real-time PCR**

Total RNA from paraffin-embedded RV tissues and cells were isolated using Recover All™ Total Nucleic Acid Isolation Kit (Invitrogen, Life technologies, California, USA) and RNeasy Mini Kit (Qiagen, Hilden, Germany) as per manufacturer’s instructions. M-MLV reverse transcriptase enzyme (Sigma, Munich, Germany) was used for reverse transcription of mRNA to cDNA and PCR’s were performed using iTaq™ Universal SYBR® Green Supermix (Bio-Rad, Munich, Germany) in Mx3000P® QPCR System machine (Stratagene, Agilent Technologies, Waldbronn, Germany). Ct values obtained were normalized to housekeeping genes beta-actin or 18S ribosomal RNA (*18s*) and relative fold change mRNA expressions were calculated to experimental controls.

**Immunofluorescence staining on human tissues**

After deparafinization and rehydration of human heart tissues, antigen-retrieval was performed in 0.01M sodium citrate buffer(pH 6.0) by microwave heating for 13 minutes. Slides were cooled down for 30 minutes and rinsed in (dulbecco’s phosphate buffered saline with 0.1% Tween 20) PBS/T 3 times, 5 minutes each. Tissues were blocked with 10% blocking serum for one hour and incubated with primary antibodies p53 antibody (D-07, MA5-14067, Thermo Fischer Scientific, Massachusetts, USA) and CD31 Antibody (ab28364, Abcam, Berlin, Germany) diluted in 2% blocking serum overnight at 4°C. Next day, slides were rinsed in PBS/T 3 times, 5 minutes each and incubated for 1 hour at room temperature with fluorescence tagged secondary antibody (Thermo Fischer Scientific, Massachusetts, USA), diluted in 2% blocking serum. The slides were rinsed in 1X PBS/T three times and mounted with Dapi-Fluoromount-G (Electron Microscopy Sciences, Pennsylvania, USA).

**Immunofluorescence staining on mouse tissues**

After deparafinization and rehydration of RV tissues, antigen-retrieval was performed in 1M EDTA buffer (pH 8.0) by microwave heating for 8 minutes. Slides were cooled down for 30 minutes and rinsed in PBS for three times, 5 minutes each. To exclude unspecific staining, tissues were blocked in 5% goat blocking buffer for one hour and incubated overnight with either fluorescence tagged Wheat Germ Agglutinin (WGA) or Isolectin GS-IB4 (IB4) at 4°C. Next day, slides were rinsed in PBS for three times, 5 minutes each. Nuclear staining was achieved by incubation with DAPI for 10 minutes. Tissues were washed in ddH2O and fixed with coverslip using ProLong Gold antifade mounting medium (Invitrogen, Thermo Scientific, Massachusetts, USA). Immunofluorescence images were taken at 20X magnification.

**Immunohistostaining**

After deparafinization and rehydration of RV tissues, slides were blocked with methanol-peroxide (3% H_2_O_2_ in methanol) for 10 minutes and rinsed in 1X PBS for 2 minutes. Tissues were blocked in Rodent block M blocking buffer (RBM961, BIOCARE MEDICAL, Pacheco, USA) and incubated in primary antibody vWF (DAKO, A0082, Santa Clara, USA) for one hour. Slides were rinsed for three times in 1XPBS, 5 minutes each and incubated with HRP polymer (POLHRP100, Zytomed systems, BERLIN, Germany) for 30 minutes. Slides were washed for two times, 3 minutes in 1X PBS and stained with DAB substrate (SK-4100, California, USA) for 5-15 minutes. For nuclear staining, tissues were counterstained with hematoxylin for 3-5 minutes.

**Terminal deoxynucleotidyl transferase dUTP nick end labeling (TUNEL) staining**

TUNEL staining’s of mouse RV tissues were performed as per manufacturer’s instructions (ROCHE, *In Situ* Cell Death Detection Kit, TMR red, Cat. No. 12156792910, Basel, Switzerland). Briefly, after deparaffinization, rehydration and antigen retrieval, slides were washed in PBS for three times, 5 minutes each. Slides were then incubated in TUNEL reaction mixture for 60 minutes at 37°C in humidified chamber. Slides were rinsed three times in PBS for 5 minutes each. To remove unspecific staining, tissues were blocked in 5% goat blocking buffer for 1 hour and counter stained with fluorescence-tagged WGA or IB4 antibodies for overnight at 4°C. Next day, slides were rinsed three times in PBS for 5 minutes each. Nuclear staining was achieved by incubation with DAPI for 10 minutes. Tissues were washed in ddH2O and fixed with coverslip using ProLong GoldAntifade Mountant (Invitrogen, Carlsbad, California, United States). Immunofluorescence images were taken at 20X magnification.

**Collagen Staining**

Paraffin embedded RV tissues were cut to 3µM sections and stained for collagen fibers with 0.1 % Sirius Red (Sirius Red F3B, Niepoetter, Bürstadt, Germany) in Picric Acid (Fluka, Neu-Ulm, Germany). Total collagen content was measured in percentage using Leica Qwin V3 image analysis software (Leica Microsystem, Wetzlar, Germany).

**List of primary and secondary antibodies for immunoblot analyses.**

| **Gene** | **Host** | **Company** | **Cat. No.** | **Dilution (in 5% BSA)** |
| --- | --- | --- | --- | --- |
| P53 (IC12) | Ms | Cell Signaling Technology, Inc., Danvers, USA | 2524S | 1:500 |
| P53 (D-07) | Rb | Thermo Fischer Scientific, Massachusetts, USA | MA5-14067 | 1:50 |
| BAX (P-19) | Rb | Santa Cruz Biotechnology, Dallas, USA | sc-526 | 1:500 |
| BCL-2 (N-19) | Rb | Santa Cruz Biotechnology, Dallas, USA | sc-492 | 1:500 |
| MDM2 | Ms | Novus Biologicals | NB600-1312 | 1:500 |
| SERCA | Rb | Badrilla Ltd. Leeds Innovation Centre | A010-20 | 1:500 |
| HO-1 (E6Z5G) | Rb | Cell Signaling Technology, Inc., Danvers, USA | 82206S | 1:500 |
| Glut1 | Rb | Merck Millipore, Burlington, USA | 07-1401 | 1:500 |
| HIF-1α | Rb | Abcam, Cambridge, United Kingdom | Ab2185 | 1:500 |
| COX2 (D5H5) | Rb | Cell Signaling Technology, Inc., Danvers, USA | 12282S | 1:500 |
| vWF | Rb | DAKO, Santa Clara, USA | A0082 | 1:100 |
| VEGF-A | Rb | Abcam, Cambridge, United Kingdom | ab46154 | 1:500 |
| Phospholamban | Rb | Badrilla Ltd. Leeds Innovation Centre | A010-12 | 1:500 |
| PGI2 synthase (PTGIS) | Ms | Santa Cruz Biotechnology, Dallas, US | sc-293247 | 1:1000 |
| Vinculin | Ms | Sigma-Aldrich, St. Louis, USA | V9131 | 1:500 |
| Amersham ECL Mouse IgG, HRP-linked whole Ab (from sheep) |  | GE Healthcare, Chicago, USA | NA931 | 1:10.000 |
| Amersham ECL Rabbit IgG, HRP-linked whole Ab (from donkey) |  | GE Healthcare, Chicago, USA | NA934 | 1:10.000 |

Secondary antibodies were always diluted in 5% non-fat dry milk in TBS/T buffer (T145.3, Carl Roth, Karlsruhe, Germany).

**List of mouse primers for qPCR analyses**

| **GENE** | **Forward primer** | **Reverse Primer** |
| --- | --- | --- |
| Vegf-a | AAAGGCTTCAGTGTGGTCTGAGA | GGTTGGAACCGGCATCTTTATC |
| Angp2 | TAGCATCAGCCAACCAGGA | AAGGACCACATGCGTCAAAC |
| Angp1 | CACGTGGAGCCGGATTTCT | ATCTGGGCCATCTCCGACTT |
| Tnnt1 | ATGGGAGCTCATTTTGGGGG | TCATCTCCCGACCAGTCTGT |
| Pln | AAAGTGCAATACCTCACTCGC | GGCATTTCAATAGTGGAGGCTC |
| Glut1 | AGCATCTTCGAGAAGGCAGG | ACAACAAACAGCGACACCAC |
| Ldha | CGTGCACTAGCGGTCTCAA | GGAGATCCATCATCTCGCCC |
| Idh2 | TCAAGTCTTCCGGTGGCTTT | ACAGATGTCATCAGGCCGAG |
| Cs | CTTGGGAGCCAAGAACTCATC | TCTGGCCTGCTCCTTAGGTAT |
| Pgc-1α | GCACGCAGCCCTATTCATTG | TGAGTCTCGACACGGAGAGT |
| Ppar-γ | TTGCTGTGGGGATGTCTCAC | AACAGCTTCTCCTTCTCGGC |
| Atp5a | ACTGCATCTACGTCGCGATT | CGCATCCGTCAGTCTCTTCA |
| Cox4i1 | GAGCACCCCAGGGTGTAGAG | TCGAAGGCACCGAAGTAG |
| Col1a1 | ACCAGCAGACTGGCAACCTCA | CATCGATGATGGGCAGGCGG |
| Col3a1 | CAA GGTCCACGAGGTGACAA | TGCCATTAGAGCCACGTTCA |
| 18S RNA | CTTCGCCATCACTGCCATTA | CGTGAGTTCTCCAGCCCTCT |

**List of rat primers for qPCR analyses**

| **GENE** | **Forward primer** | **Reverse Primer** |
| --- | --- | --- |
| Bax | TAGCAAACTGGTGCTCAAGG | AGCCACAAAGATGGTCACTG |
| Mdm2 | GTAGCATTGTTTACAGCAGCC | CAACCATTTTTAGGCCGCC |
| Ho-1 | TTAAGCTGGTGATGGCCTCC | GTGGGGCATAGACTGGGTTC |
| Cox2 | TGACTGTACCCGGACTGGAT | TGGATTGAATTCGAAGGAAGGGA |
| Glut1 | GAGTGTACTGTGGCCTGACC | GTCTAAGCCGAACACCTGGG |
| Angp1 | TCGCTGCCATTCTGACTCAC | GGCCATCTCCGACTTCATGT |
| Angp2 | CATGATGTCATCGCCCGACT | CCTCCACCCATGTCCATGTC |
| Vegfa | CAAACCTCACCAAAGCCAGC | TTCTCCGCTCTGAACAAGGC |
| Hif-1α | GCCACACTGCGGCTGGTTAC | GCCACACTGCGGCTGGTTAC |
| beta-actin | CCCGCGAGTACAACCTTCT | CGTCATCCATGGCGAACT |

1. Schluter KD, Schreiber D. Adult ventricular cardiomyocytes: isolation and culture. Methods Mol Biol. 2005;290:305-14. doi: 10.1385/1-59259-838-2:305. PubMed PMID: 15361670.

2. Novoyatleva T, Schymura Y, Janssen W, Strobl F, Swiercz JM, Patra C, et al. Deletion of Fn14 receptor protects from right heart fibrosis and dysfunction. Basic Res Cardiol. 2013;108(2):325. doi: 10.1007/s00395-012-0325-x. PubMed PMID: 23325387; PubMed Central PMCID: PMCPMC3597271.
