# Supplementary material for: Effect of p53 activation on experimental right ventricular hypertrophy
Source: PLoS One. 2020 Jun 19;15(6):e0234872. doi: 10.1371/journal.pone.0234872 (PMC7304610; doi:10.1371/journal.pone.0234872)
Supplement: S1 Raw images — (PDF) [file pone.0234872.s006.pdf]

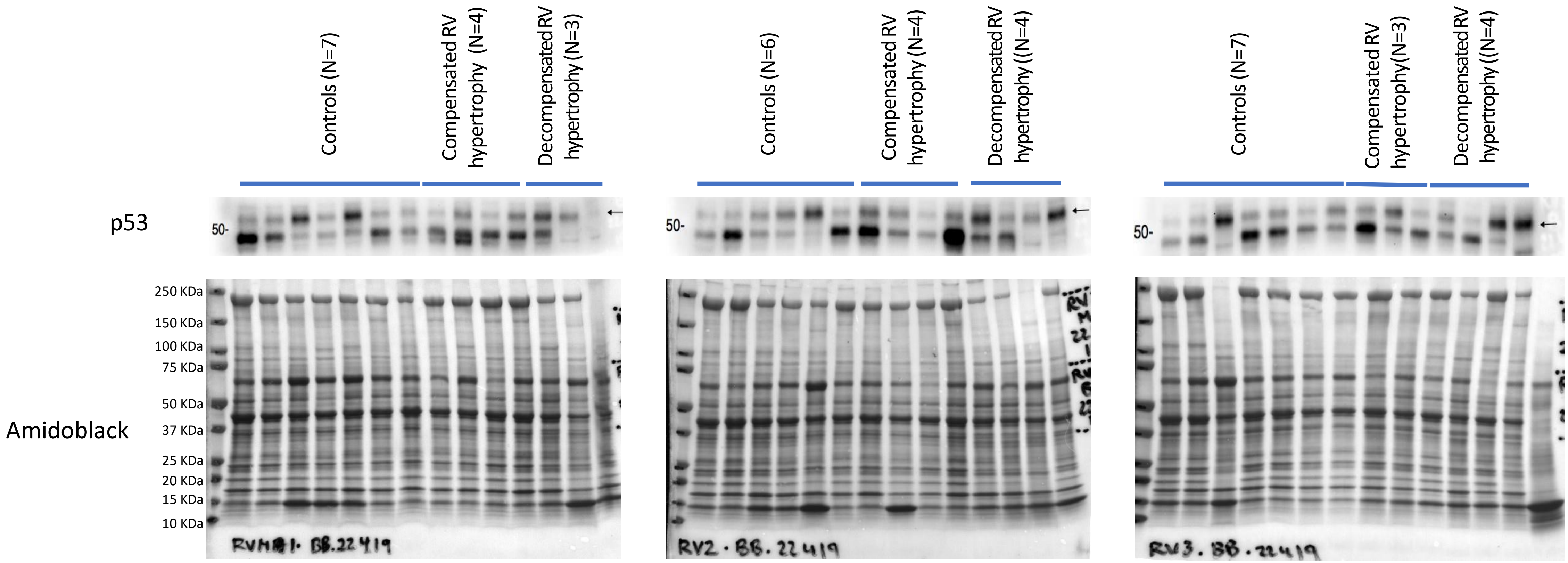

Original blots for Fig.1

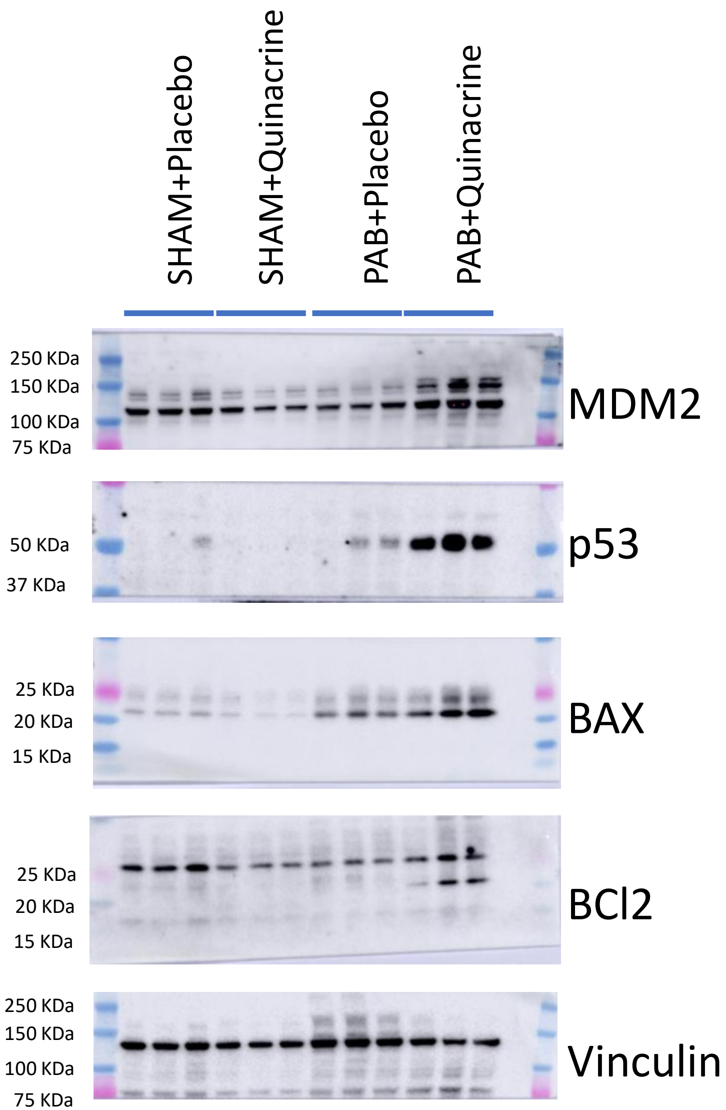

Original blots for Fig.3

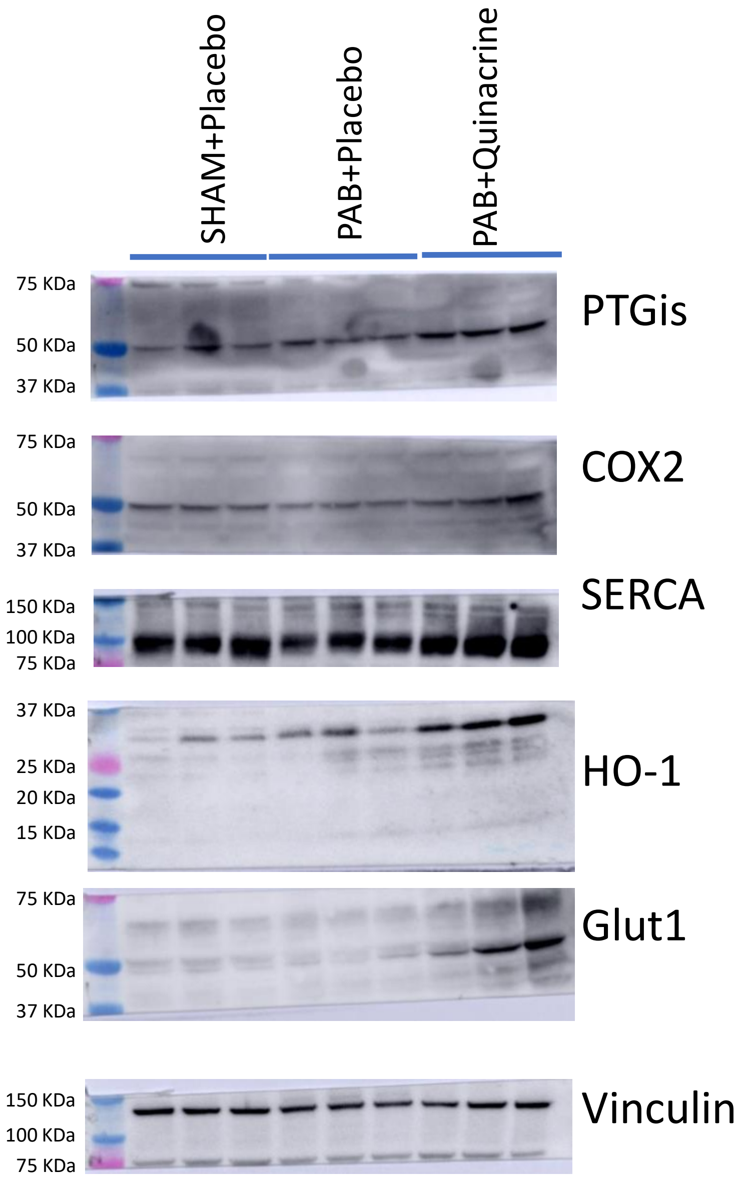

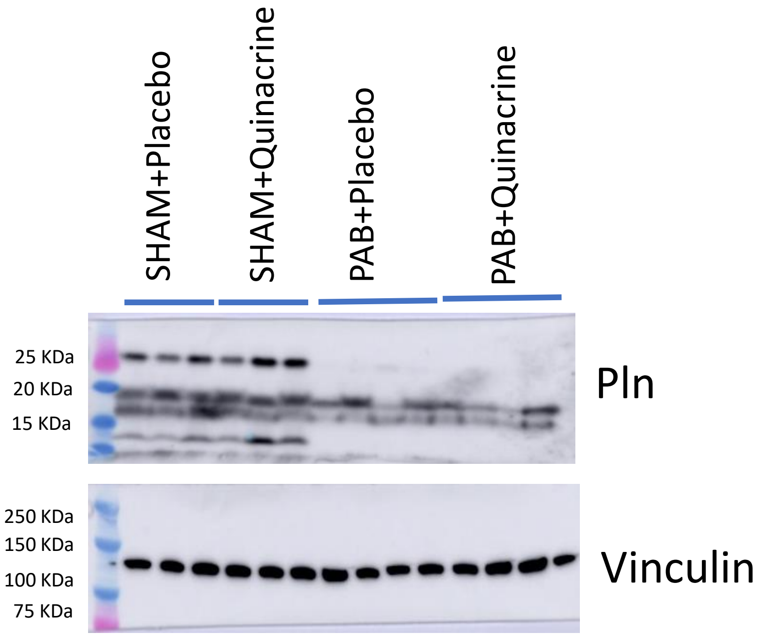

Original blots for Fig.4

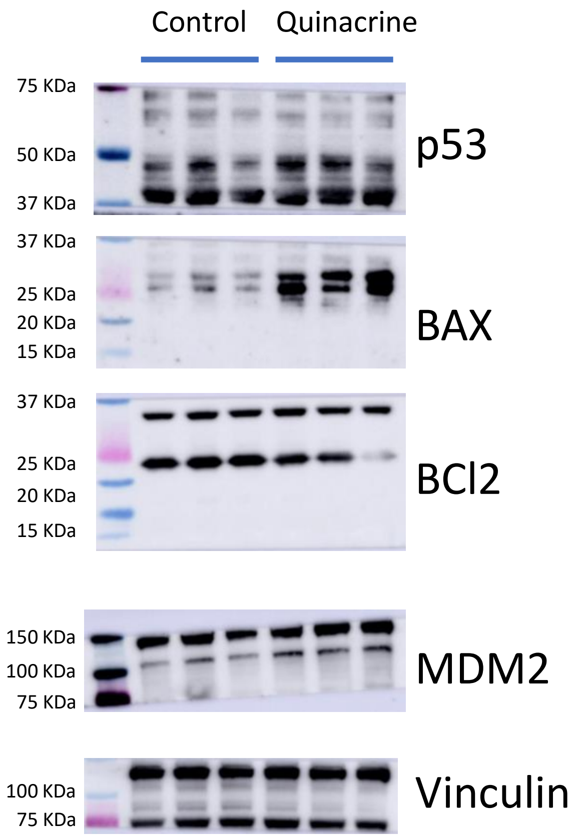

RV cardiomyocytes

Original blots for Fig 5

A

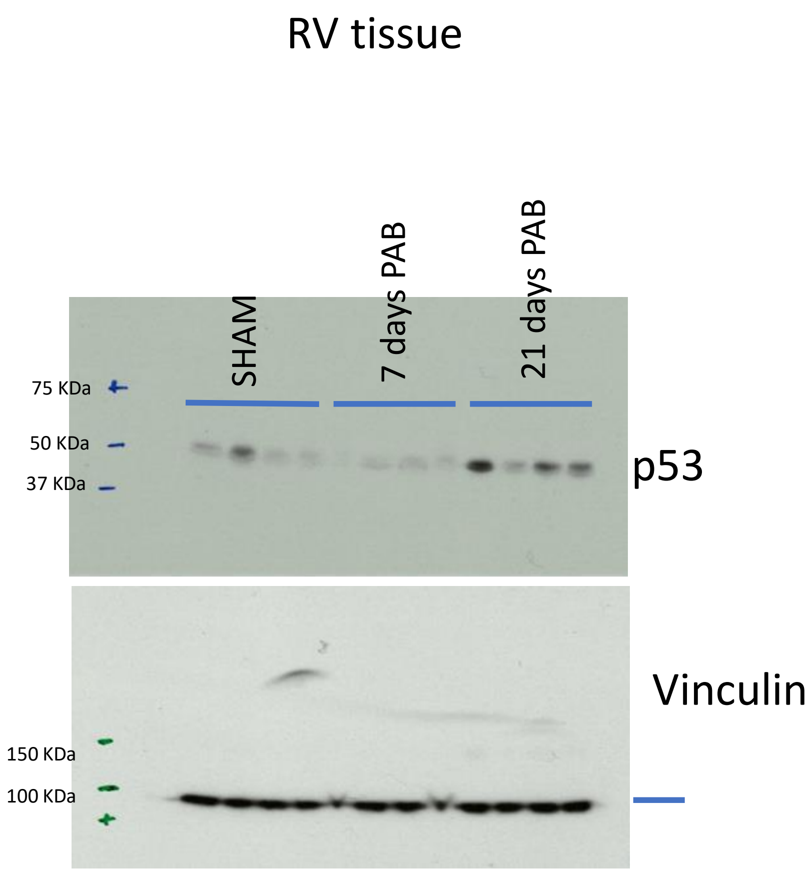

D

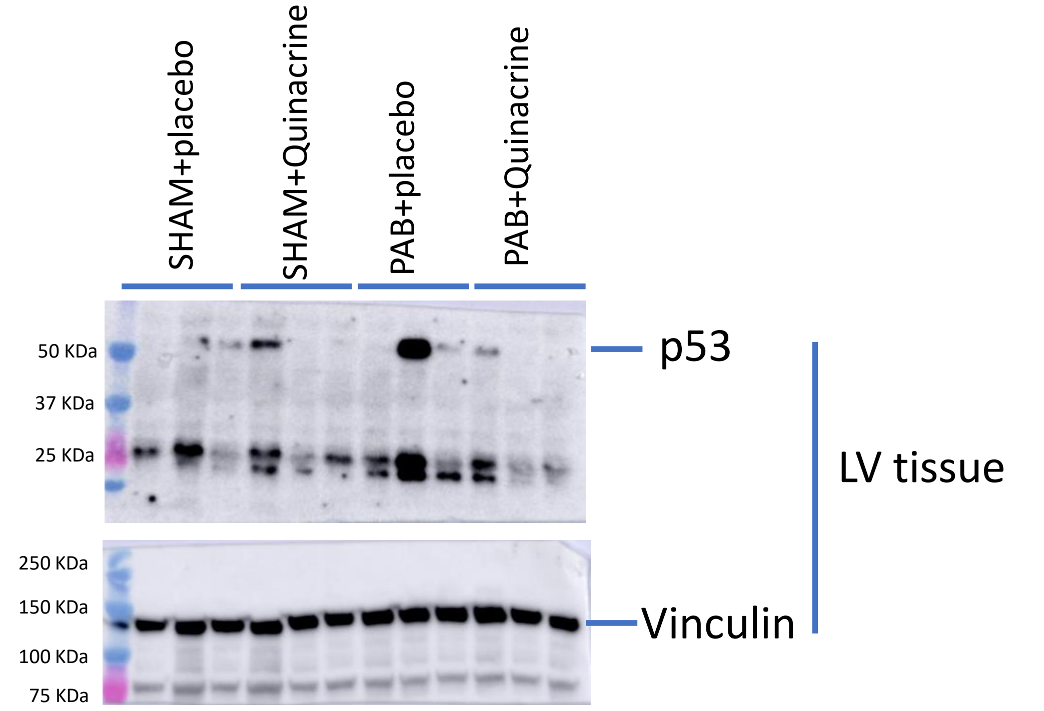

E

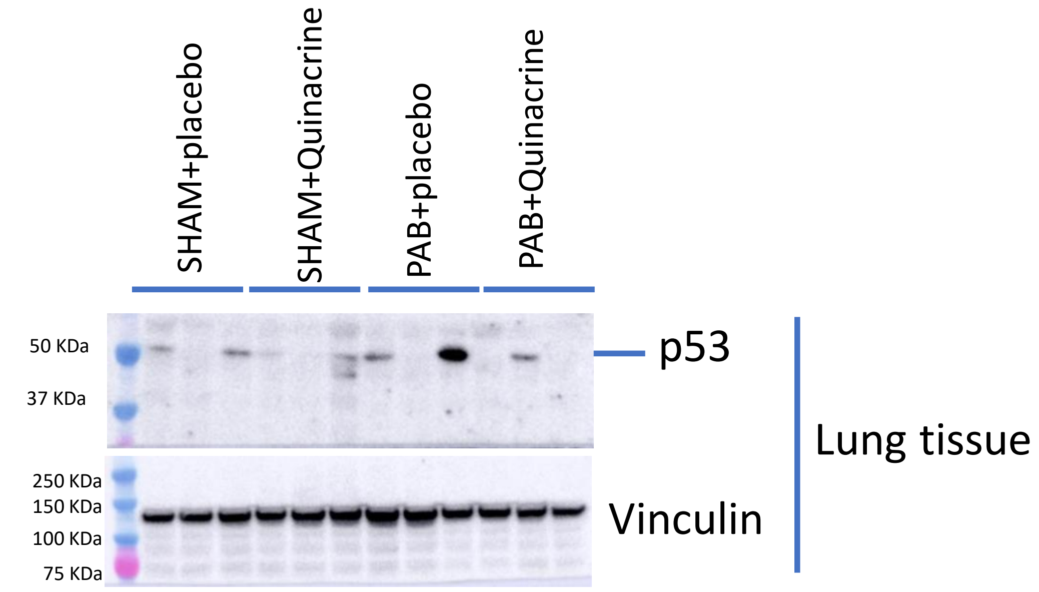

Original blots for supplemental Fig 1E

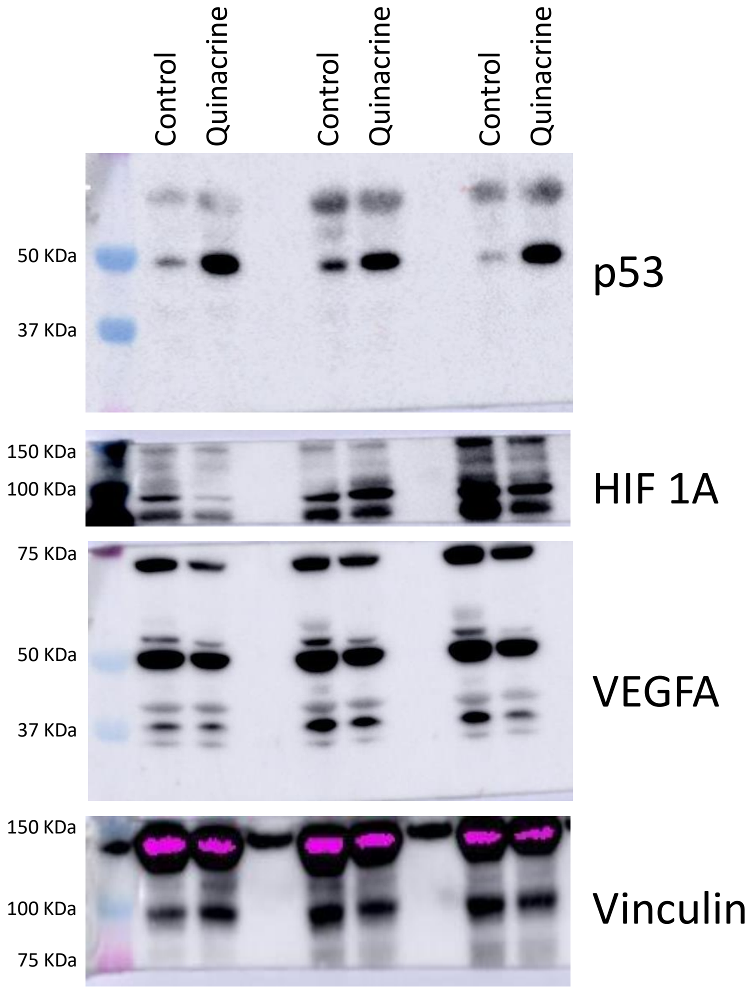

Original blots for Supplemental fig 2E
